# Mechanistic and Kinetic Study on Self-/Cross- Condensation of PCTA/DT Formation Mechanisms from Three Types of Radicals of 2,4-Dichlorothiophenol

**DOI:** 10.3390/ijms20112623

**Published:** 2019-05-28

**Authors:** Hetong Wang, Chenpeng Zuo, Siyuan Zheng, Yanhui Sun, Fei Xu, Qingzhu Zhang

**Affiliations:** 1 Environment Research Institute, Shandong University, Qingdao 266237, China; Kishi_Wang@163.com (H.W.); zuochenpeng@126.com (C.Z.); zhengsiyuan1991@126.com (S.Z.); zqz@sdu.edu.cn (Q.Z.); 2 College of Environment and Safety Engineering, Qingdao University of Science & Technology, Qingdao 266042, China; sunyh0532@126.com; 3 Shenzhen Research Institute, Shandong University, Shenzhen 518057, China

**Keywords:** 2,4-dichlorothiophenoxy radical, 2-sulfydryl-3,5-dichlorophenyl radical, 3,5-dichlorothiophenoxyl diradical, PCTA/DTs, formation mechanism, rate constants

## Abstract

Chlorothiophenols (CTPs) are known to be key and direct precursors of polychlorinated thianthrene/dibenzothiophenes (PCTA/DTs). Self/cross-coupling of the chlorothiophenoxy radicals (CTPRs), sulfydryl-substituted phenyl radicals and thiophenoxyl diradicals evolving from CTPs are initial and important steps for PCTA/DT formation. In this study, quantum chemical calculations were carried out to investigate the homogenous gas-phase formation of PCTA/DTs from self/cross-coupling of 2,4-dichlorothiophenoxy radical (R1), 2-sulfydryl-3,5-dichlorophenyl radical (R2) and 3,5-dichlorothiophenoxyl diradical (DR) at the MPWB1K/6-311+G(3df,2p)//MPWB1K/6-31+G(d,p) level. The rate constants of crucial elementary steps were deduced over 600–1200 K, using canonical variational transition state theory with a small curvature tunneling contribution. For the formation of PCTAs, the S•/σ-C• condensation with both thiophenolic sulfur in one radical and *ortho* carbon in the other radical bonded to single electron is the most efficient sulfur-carbon coupling mode, and the ranking of the PCTA formation potential is DR + DR > R2 + DR > R1 + DR > R1 + R2 > R1 + R1. For the formation of PCDTs, the σ-C•/σ-C• coupling with both *ortho* carbon in the two radicals bonded to single electron is the energetically favored carbon-carbon coupling mode, and the ranking of the PCDT formation potential is: R2 + DR > R2 + R2 > R1 + DR > R1 + R2 > R1 + R1. The PCTA/DTs could be produced from R1, R2 and DR much more readily than PCDD/DFs from corresponding oxygen substituted radicals.

## 1. Introduction

Polychlorinated thianthrene/dibenzothiophenes (PCTA/DTs) are two groups of sulfur-substituted structural analogues of the polychlorinated dibenzo-p-dioxin/dibenzofurans (PCDD/DFs), which are persistent, lipophilic and considered to be dioxin-like compounds [1]. In general, PCTA/DTs exhibit less toxicity than PCDD/DFs with the toxicity equivalent factor (TEF) of 2,3,7,8-TeCTA and 2,3,7,8-TeCDT as 0.01 and 0.001, respectively [2]. Kopponen et al. found that the potency of 2,3,7,8-tetrachlorothianthrene (TeCTA) and 2,3,7,8-tetrachlorodibenzothiophene (TeCDT) to induce aryl hydrocarbon hydroxylase (AHH) is reported to be approximately 1/400 that of 2,3,7,8-TeCDD [3]. Owning to the difference of chlorine substitution positions and numbers, PCTA/DTs are identified as having 75 congeners of PCTAs and 135 congeners of PCDTs. Among them, 2,4,6,8-TeCDT is the most widely detected PCTA/DTs [4,5,6,7,8,9,10,11,12]. For example, Buser et al. reported that 2,4,6,8-TCDT constitutes approximately 95% of the total TCDT in the Passaic River [6]. PCTA/DTs have similar and even much higher concentration in some regions and bioaccumulation potential than PCDD/DFs [4,13], which have been detected in the different environmental samples, including soil and sediment [11,14], pulp bleaching [1], incineration of municipal waste [15,16], wastes from petroleum refineries [17] and petroleum spills [18]. For example, Rappe et al. reported the presence of PCDTs in emissions from iron and steel sintering plants and foundries at approximately the same concentrations as PCDFs [8]. Cai et al. found that blue crabs included more 2,4,6,8-TeCDT than 2,3,7,8-TeCDT in their muscle and hepato-pancreatic tissues [19]. Pruell et al. observed that the mean concentrations of 2,4,6,8-TeCDT (3680 ng/kg) in Passaic River sediments were 5-10 times higher than that of 2,3,7,8-TeCDD (656 ng/kg) and 2,3,7,8-TeCDF (334 ng/kg) [11]. Thus, considering the serious environmental pollution and wide concern by scientific researchers of these toxic substances, it is significant to clarify the formation mechanism of PCTA/DTs under combustion and thermal processes to hinder their harm to humans and the environment.

PCTA/DTs were never intentionally synthesized for commercial purposes, but are formed as byproducts from a variety of combustion and thermal processes such as municipal and hazardous waste incinerators as well as industrial incinerators [6,7]. The most direct route to the formation of PCTA/DTs is the gas-phase reaction from chemical precursors. Among different precursors, chlorothiophenols (CTPs) are the most direct precursors of PCTA/DTs [20,21,22], which have been widely used in large quantities in various chemical industries, such as in manufacturing of dyes, insecticides, printing inks, pharmaceuticals, and polyvinyl chloride [23]. Similar to the formation of PCDD/DFs from chlorophenol (CP) precursors, the gas-phase formation of PCTA/DTs from CTP precursors was also proposed involving radical-radical coupling of two CTPRs and radical-molecule recombination of CTPR and CTP [21,22,24,25,26,27,28]. Dar et al. have shown that radical-radical coupling is more competitive thermodynamically than radical-molecule recombination for PCTA/DT formation [21,22,24]. The principal work located the radical-radical coupling to form PCTA/DTs on the condensation of two CTPRs [21,22,24]. One study in our group has shown the homogeneous gas-phase formation mechanism of PCTA/DTs from the coupling of 2,4-dichlorothiophenoxy radicals (2,4-DCTPR) and coupling of 2,4,6-trichlorothiophenoxy radicals (2,4,6-TCTPR) [29]. Recently, Yu et al. have proposed that besides CTPRs, various similar radicals including sulfydryl-substituted phenyl radicals and thiophenoxyl diradicals may also be key intermediates in the formation process of PCTA/DTs from CTPs [30]. The homogeneous gas-phase self- and cross- dimerization of these radicals as well as CTPRs could be the initial and important step for the formation of PCTA/DTs [30]. Although sulfydryl-substituted phenyl radicals and thiophenoxyl diradicals have not yet been identified in combustion and thermal processes, their oxygenated counterparts phenyl radicals and phenoxyl diradical have been detected and proposed to be highly active and have great potential for the gas-phase formation of PCDD/DFs [31,32,33,34,35]. A theoretical study by Pan et al. has also proved the formation feasibility of phenyl radicals and phenoxyl diradicals and their energetically favorable contribution to PCDD/DF formation [36]. The structure similarity and high concentration correlation between PCTA/DTs and PCDD/DFs in the environmental samples unsurprisingly revealed their analogous formation mechanism under pyrolysis or combustion conditions [15,37,38]. These experimental results have urged us to conjecture similar operating reactions for PCTA/DT formation from CTPRs, sulfydryl-substituted phenyl radicals and thiophenoxyl diradicals as precursors or key intermediates based on PCDD/DF formation from CPRs, phenyl radicals and phenoxyl diradicals as precursors in essentially all proposed pathways [36]. Under the pyrolysis or combustion conditions, CTPRs can be formed through loss of the thiophenoxyl-H, sulfydryl-substituted phenyl radicals can be derived from dissociation of the Cl or H atom combining with the carbon in the adjacent position of the carbon with -SH group, and the thiophenoxyl diradical are sourced from the loss of both the triophenoxyl-H and the *ortho*-substituted Cl or H atom via unimolecular, bimolecular, or possibly other low-energy pathways (including heterogenous reactions). The bimolecular reactions include attack by H, OH, O(^3^P), or Cl under high-temperature oxidative conditions. Yu’s study mainly focused on the formation of CTPR, sulfydryl-substituted phenyl radical and thiophenoxyl diradical from the abstraction of 2-chlorophenol (2-CP) by H, and studied the subsequent PCTA/DT formation pathways by two representative radical-radical coupling reactions [30]. Therefore, a detailed PCTA/DT formation mechanism from self-/cross- coupling of CTPR, sulfydryl-substituted phenyl radical and thiophenoxyl diradical are needed for further investigation to verify the contributions to PCTA/DT formation of these radicals.

In this paper, we present a systematic theoretical study on the PCTA/DT formation mechanism from 2,4-dichlorothiophenol (2,4-DCTP) as precursor by the self-/cross- condensation of 2,4-dichlorothiophenoxy radical (R1), 2-sulfydryl-3,5-dichlorophenyl radical (R2) and 3,5-dichlorothiophenoxyl diradical (DR). 2,4-DCTP was selected as the model because it has the minimum number of Cl atoms to form 2,4,6,8-TeCDT, which was the most widely detected and important PCTA/DTs in the environment. In addition, as part of our ongoing work on PCTA/DT from R1 + R1 coupling, we compared the R1 + R2, R1 + DR, R2 + DR, R2 + R2 and DR + DR coupling pathways in this study with the R1 + R1 coupling routes in our previous work [29], and sorted all these coupling reactions to confirm their contribution to the formation of PCTA/DTs. Furthermore, the kinetic data and rate constants were evaluated over a wide temperature range of 600–1200 K and fitted into Arrhenius formulas to improve and optimize PCTA/DT formation mathematic models.

## 2. Results

The reliability and accuracy of the MPWB1K/6-311+G(3df,2p)// MPWB1K/6-31+G(d,p) level for the geometries, frequencies and energy calculation in this study have been confirmed in our previous works on PCTA/DT formation with 2,4-DCTP as the precursor [29,39]. The data was compared with data from Yu and Pan calculated at the BB1K/6-311+G(3df,2p)//BB1K/6-311G(d,p) level [30,36]. Several typical elementary reactions in this study were calculated at the same level shown in Table S1 of Supplementary Materials. The calculated results in this study agree well with the BB1K/6-311+G(3df,2p)//BB1K/6-311G(d,p) values (the relative deviation remains within 0.48 kcal/mol for potential barriers and 1.19 kcal/mol for reaction heats). The imaginary frequencies, the zero-point energies and the total energies for the transition states involved in the formation of PCTA/DTs from R1, R2 and DR are shown in Table S2. Cartesian coordinates for the reactants, intermediates, transition states and products involved in formation of PCTA/DTs from R1, R2 and DR are depicted in Tables S4 and S5.

### 2.1. Formation of 2,4-Dichlorothiophenoxy Radical R1, 2-Sulfydryl-3,5-Dichlorophenyl Radical R2 and 3,5-Dichlorothiophenoxyl Diradical DR

**Figure 1:**
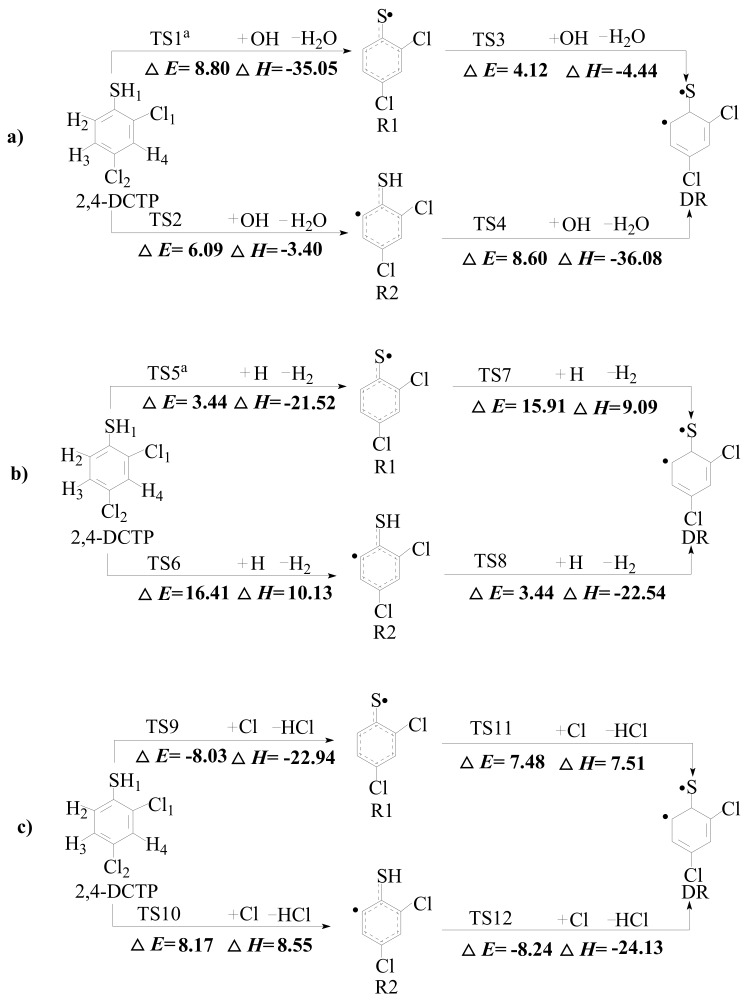
Schematic formation of various radicals from the reactions of 2,4-DCTP with OH (**a**), H (**b**) and Cl (**c**) with the potential barriers Δ*E* (in kcal/mol) and reaction heats Δ*H* (in kcal/mol). (^a^ Reproduced with permission from Xu et al. [39]).

**Figure 2:**
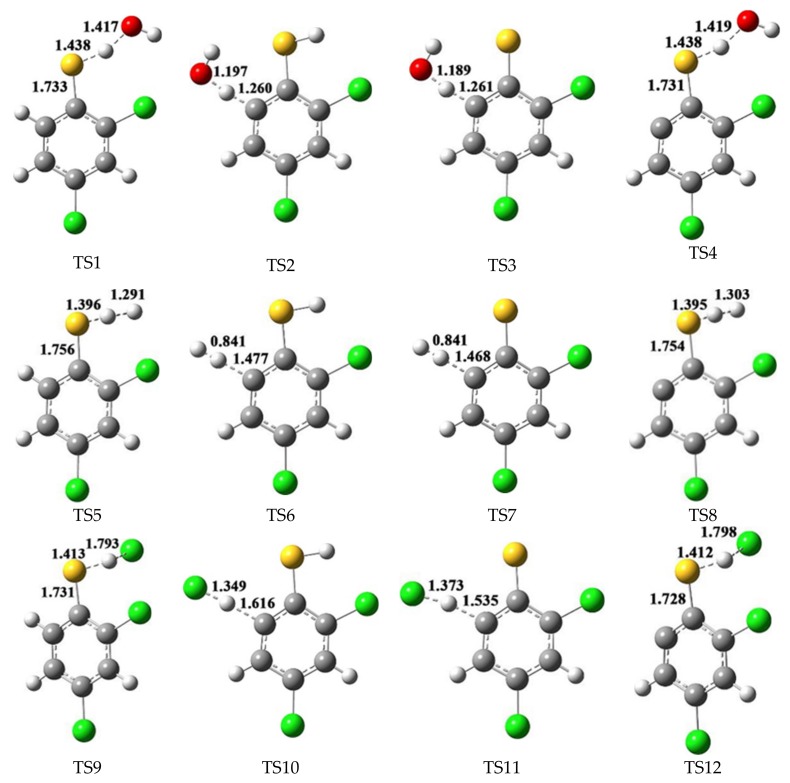
MPWB1K/6-31+G(d,p) optimized transition state geometries for reactions between 2,4-DCTP with OH, H and Cl radicals. The bond distances are given in Å. Green, gray, yellow, red and white balls denote Cl, C, S, O and H atoms, respectively.

The formation of 2,4-dichlorothiophenoxy radical (R1), 2-sulfydryl-3,5-dichlorophenyl radical (R2) and 3,5-dichlorothiophenoxyl diradical (DR) which derive from 2,4-DCTP is the initial and key step in the formation of PCTA/DTs. These radicals may be generated by the bimolecular reaction of 2,4-DCTP with the active radicals H, OH and Cl which exist abundantly in combustion and thermal processes. The calculated potential barriers (Δ*E*) and the reaction heats (Δ*H*) at the MPWB1K/6-311+G(3df,2p)//MPWB1K/6-31+G(d,p) level involved in the formation of R1, R2 and DR radicals from 2,4-DCTP abstracted by the OH, H and Cl are given in the Figure 1a–c. In Figure 1, data of 2,4-DCTP thiophenoxyl-H abstraction by OH and H were cited from our previous studies [39]. All the optimized transition state geometries for 2,4-DCTP abstracted by OH, H and Cl radicals are shown in Figure 2.

In Figure 1, the sulfydryl group H atom in 2,4-DCTP is labeled as H1, the extra three H atom at the benzene ring are numbered as H2-H4, and two Cl atoms at the benzene ring are numbered as Cl1 and Cl2. The R1 radical can be produced through loss of the thiophenoxyl-hydrogen (H1) abstracted by H, OH and Cl radicals. The R2 radical can be formed through elimination of the thiophenyl-hydrogen (H2). As mentioned by Yu et al. [30], five chlorinated thiophenyl radicals can be formed by abstracting H2–H4 and Cl1–Cl2, but only two chlorinated thiophenyl radicals losing *ortho* H or Cl (H2 or Cl1) can further react to form PCTA/DTs [30,40]. In addition, chlorinated thiophenyl radical losing H2 contribute to the formation 2,4,6,8-TeCDT, which must be produced from 2,4-DCTP without Cl loss. Thus, only one chlorinated thiophenyl radical R2 formed by abstracting H2 is further studied in this paper. The DR radical can be generated by fission of both thiophenoxyl-hydrogen and thiophenyl-hydrogen (H1 and H2). 

### 2.2. Formation of PCTAs from R1, R2 and DR

**Figure 3:**
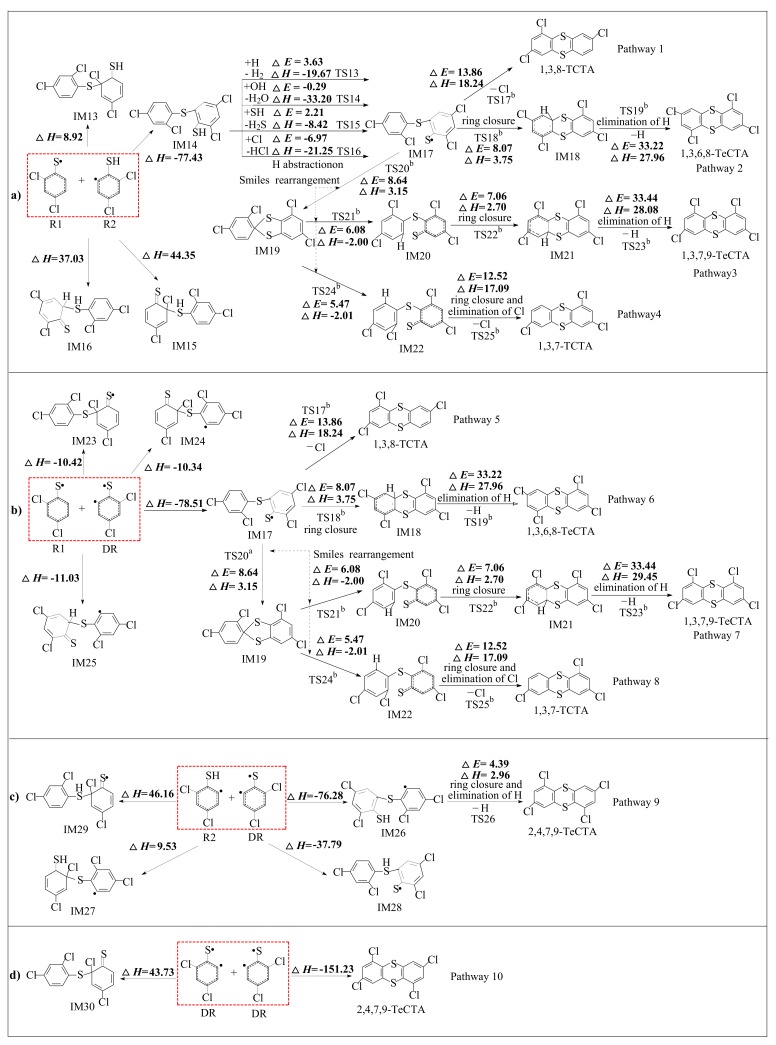
Polychlorinated thianthrene (PCTA) formation routes embedded with the potential barriers Δ*E* (in kcal/mol) and reaction heats Δ*H* (in kcal/mol) from self- and cross-couplings of R1 + R2 (**a**), R1 + DR (**b**), R2 + DR (**c**), and DR + DR (**d**). Δ*H* is calculated at 0 K. (^b^ Reproduced with permission from Xu et al. [29]).

Figure 3 illustrates schematically the proposed reaction pathways for the formation of PCTAs from radical-radical cross-condensation R1 + R2 (in Figure 3a), R1 + DR (in Figure 3b) and R2 + DR (in Figure 3c), and self-condensation of DR + DR (in Figure 3d). The potential barriers Δ*E* (in kcal/mol) and reaction heats Δ*H* (in kcal/mol) are calculated at the MPWB1K/6-311+G(3df,2p)//MPWB1K/6-31+G(d,p) level. As shown in Figure 3, ten possible pathways, denoted as pathways 1–10, are postulated, resulting in five PCTA congeners (1,3,8-TCTA, 1,3,7-TCTA, 1,3,6,8-TeCTA, 1,3,7,9-TeCTA and 2,4,7,9-TeCTA). Among them, pathway 1 to pathway 8 from R1 + R2 and R1 + DR converge to a common intermediate, IM5, which also lies in the self-coupling of 2,4-dichlorothiophenol (R1 + R1) in our previous paper [29].

In R1 + R2 route in Figure 3a, the cross-coupling of R1 + R2 leads to the formation of IM1, IM2, IM3 and IM4 from four kinds of sulfur-carbon coupling modes: (1) The coupling of the thiophenolic sulfur bonded to single electron with the *ortho* carbon bonded to chlorine of the chlorothiophenyl radical (S•/σ-CCl for short), (2) the coupling of the thiophenolic sulfur bonded to single electron with the *ortho* carbon bonded to single electron of the chlorothiophenyl radical (S•/σ-C• for short), (3) the coupling of the thiophenolic sulfur bonded to hydrogen of the chlorothiophenyl radical with the *ortho* carbon bonded to chlorine of the chlorothiophenoxy radical (SH/σ-CCl for short), and (4) the coupling of the thiophenolic sulfur of the chlorothiophenyl radical bonded to hydrogen with the *ortho* carbon bonded to hydrogen of the chlorothiophenoxy radical (SH/σ-CH for short). And it can be seen from Figure 3a that pathway 1 involves three elementary steps: (1) sulfur-carbon coupling; (2) H abstraction and (3) ring closure and intra-annular elimination of Cl. The ring closure and intra-annular elimination of Cl occur in a one-step reaction and are the concerted reactions. From Figure 3a, pathway 2 contains four elementary pathways: (1) sulfur-carbon coupling; (2) H abstraction; (3) ring closure and (4) intra-annular elimination of H. The ring closure and elimination of H steps occur separately. Pathway 3 and Pathway 4 include two smiles rearrangement steps before ring closure step compared to pathway 2 and Pathway 1 and has six elementary steps and five elementary steps, respectively.

In Figure 3b, the cross-coupling reaction of R1 + DR also yield four intermediates (IM5, IM11, IM12 and IM13) from four kinds of coupling modes (S•/σ-C•coupling, two kinds of S•/σ-CCl coupling and S•/σ-CH coupling), respectively. From Figure 3c, there are four kinds of cross-coupling modes of R2 + DR (S•/σ-C• coupling, S•/σ-CCl coupling, SH/σ-CCl coupling and SH/σ-C• coupling), which generates IM14, IM15, IM16 and IM17. Owning to the symmetry of self-coupling of DR + DR, there are only two sulfur-carbon coupling modes in Figure 3d: S•/σ-C• couplings and S•/σ-CCl couplings. Besides the ten pathways, we also studied the PCTA formation of R2 + R2 coupling in Figure S1 of Supplementary Materials. There are two sulfur-carbon coupling modes in R2 + R2 coupling, which includes SH/σ-CCl coupling and SH/σ-C• coupling.

### 2.3. Formation of PCDTs from R1, R2 and DR

**Figure 4:**
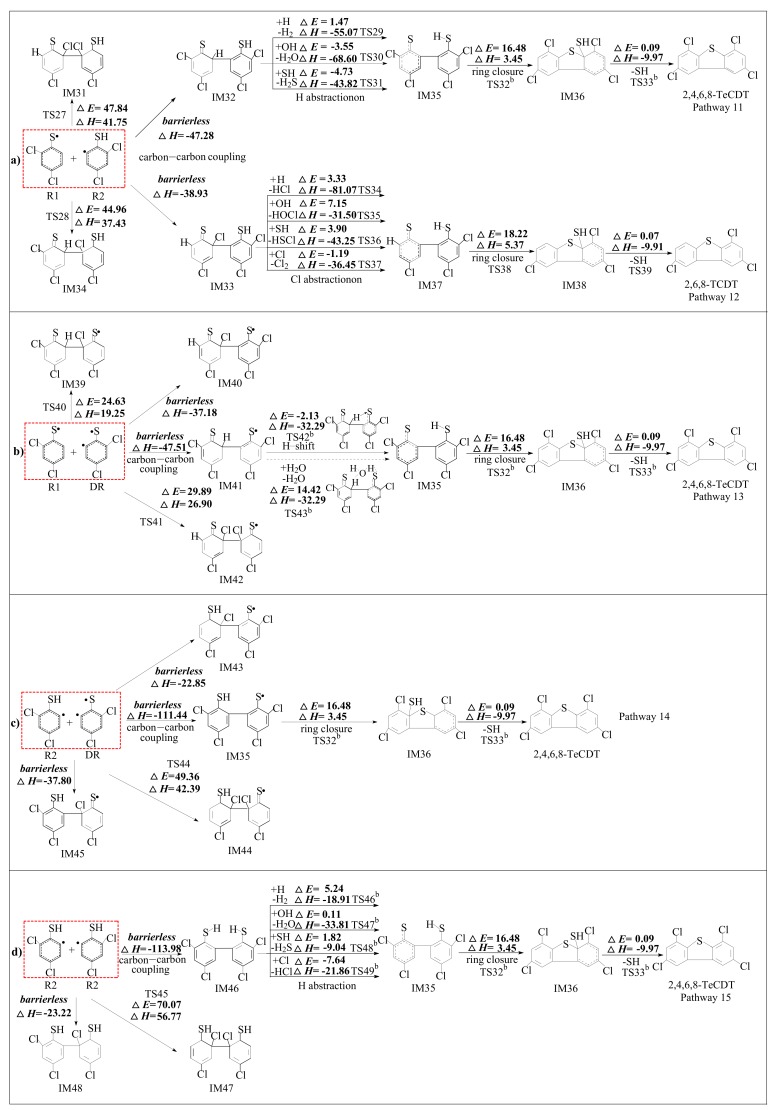
Polychlorinated dibenzothiophene (PCDT) formation routes embedded with the potential barriers Δ*E* (in kcal/mol) and reaction heats Δ*H* (in kcal/mol) from self- and cross-couplings of R1 + R2 (**a**), R1 + DR (**b**), R2 + DR (**c**), and R2 + R2 (**d**). Δ*H* is calculated at 0 K. (^b^ Reproduced with permission from Xu et al. [29]).

**Figure 5:**
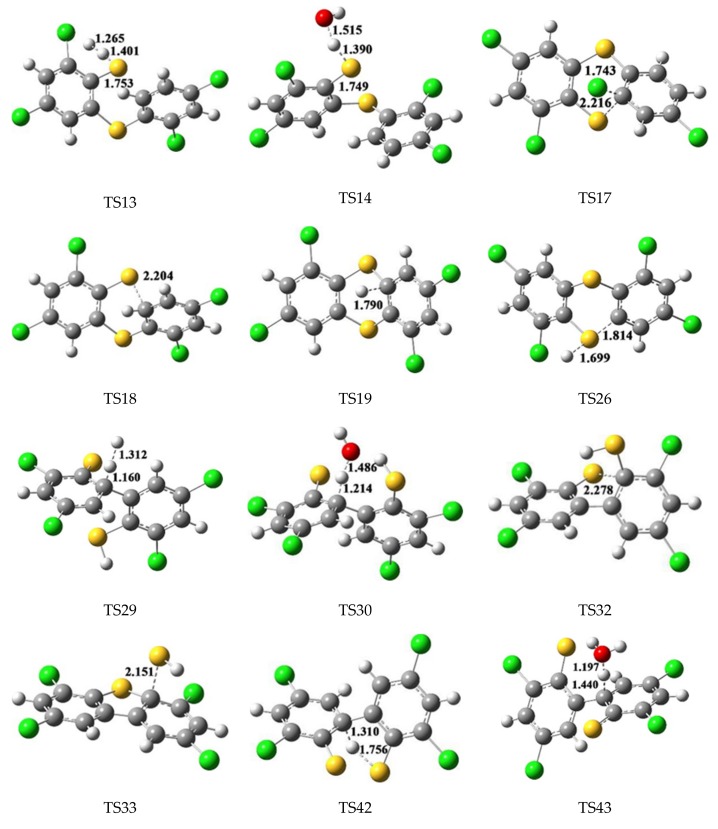
MPWB1K/6-31+G(d,p) optimized transition state geometries in the formation of PCTA/DTs. The bond distances are given in Å. Green, gray, yellow, red and white balls denote Cl, C, S, O and H atoms, respectively.

Figure 4 depicts the homogeneous gas-phase formation mechanisms of PCDTs from radical-radical cross-condensation R1 + R2 (in Figure 4a), R1 + DR (in Figure 4b) and R2 + DR (in Figure 4c), and self-condensation of R2 + R2 (in Figure 4d). Five formation pathways are postulated to explain two PCDT congeners (2,4,6,8-TeCDT and 2,6,8-TCDT) formations in Figure 4. Among them, pathway 11, 13, 14 and 15 from R1 + R2, R1 + DR, R2 + DR and R2 + R2 converged to a common intermediate, IM23, which are identified as the pre-intermediate of 2,4,6,8-TeCDT. In our previous paper, self-coupling of 2,4-dichlorothiophenol (R1 + R1) can also form IM23, which creates analogously consequent reactions to form 2,4,6,8-TeCDT [29]. Pathway 12 are proposed to interpret the formation of 2,6,8-TCDT, and has the identical pre-intermediate IM25 compared with the 2,6,8-TCDT formation pathway from self-coupling of 2,4-dichlorothiophenol (R1 + R1) [29]. Several typical optimized transition state geometries in the formation of PCTA/DTs are shown in Figure 5.

In Figure 4a, the coupling reaction of R1 + R2 leads to the formation of IM19, IM20, IM21 and IM22 from four kinds of coupling modes: (1) The coupling of *ortho* carbon bonded to chlorine of R1 with the *ortho* carbon bonded to chlorine of R2 (σ-CCl/σ-CCl for short); (2) the coupling of *ortho* carbon bonded to hydrogen of R1 with the *ortho* carbon bonded to single electron of R2 (σ-CH/σ-C• for short); (3) the coupling of *ortho* carbon bonded to chlorine of R1 with the *ortho* carbon bonded to single electron of R2 (σ-CCl/σ-C• for short) and (4) the coupling of *ortho* carbon bonded to hydrogen of R1 with the *ortho* carbon bonded to chlorine of R2 (σ-CH/σ-CCl for short). In Figure 4a, both pathways 11 and 12 involve four elementary steps: (1) carbon-carbon coupling; (2) H/Cl abstraction; (3) ring closure and (4) elimination of SH. The H/Cl abstraction step can proceed due to being abstracted by H, OH, SH and Cl radicals.

In Figure 4b, the coupling reactions of R1 + DR also produce four intermediates (IM27, IM28, IM29 and IM30) from four kinds of coupling modes. The formation of IM27 is from σ-CH/σ-CCl coupling and formation of IM30 is from σ-CCl/σ-CCl coupling. And the formation of IM28 is from σ-CCl/σ-C• coupling and formation of IM29 is from σ-CH/σ-C• coupling. In Figure 4b, pathway 13 is comprised of four elementary steps: (1) carbon-carbon coupling; (2) H shift (without and with water); (3) ring closure and (4) elimination of SH. The rate-determining step occurs in the ring closure step. As shown in Figure 4b, water molecular can participate actively in the H shift step though forming a seven-membered water bridge ring, which accepts the H from the aromatic ring and simultaneously donates another H atom to the S atom. The R2 + DR route involves four kind of coupling modes resulting in the formation of IM23, IM31, IM32 and IM33 in Figure 4c. Pathway 14 covers three elementary processes to form 2,4,6,8-TeCDT: (1) carbon-carbon coupling; (2) ring closure and (3) elimination of SH. And there exists a new coupling mode (the coupling of *ortho* carbon bonded to single electron of R2 with the *ortho* carbon bonded to single electron of DR (σ-C•/σ-C• for short)), which leads to the formation of IM23. The R2 + R2 can proceed to self-coupling, which results in IM34, IM35 and IM36 as depicted in Figure 4d.

### 2.4. Rate Constant Calculations

The kinetic parameters, such as the pre-exponential factor, the activation energy, and the rate constants of the elementary reactions of PCTA/DT formation are significant for constructing the formation kinetic model to predict the potential emissions and poisonousness to the environment. Thus, the rate constants of the crucial elementary reactions in the formation of the PCTA/DTs from R1, R2 and DR were calculated by using canonical variational transition state theory (CVT) with small-curvature tunneling (SCT) contribution methods in the temperature range of 600–1200 K relevant to real situations at high temperature PCTA/DT formation conditions [41,42,43,44]. The results are shown in Table S3 of Supplementary Materials.

To be applied more effectively, the CVT/SCT rate constants were fitted, and Arrhenius formulas are given in Table 1 for the elementary reactions involved in the formation of PCTA/DTs from R1, R2 and DR. The pre-exponential factor, the activation energy, and the rate constants can be obtained from these Arrhenius formulas.

## 3. Discussion

### 3.1. Formation of 2,4-Dichlorothiophenoxy Radical R1, 2-Sulfydryl-3,5-Dichlorophenyl Radical R2 and 3,5-Dichlorothiophenoxyl Diradical DR

In Figure 1, for the formation of R1, the potential barrier of H1 abstraction of 2,4-DCTP by OH, H and Cl radicals is 8.80 kcal/mol, 3.44 kcal/mol and −8.03 kcal/mol, respectively. Similarly, for the formation of R2, the potential barrier of H2 abstraction of 2,4-DCTP abstracted by OH, H and Cl radicals is 6.09 kcal/mol, 16.41 kcal/mol and 8.17 kcal/mol, respectively. Apparently, the formation of R1 through H and Cl abstraction involves lower potential barriers than that of R2 through H and Cl, respectively. For the OH abstraction reactions, the potential barrier of the formation of R2 is slightly lower than R1. In addition, the formation of R1 abstracted by OH, H and Cl (−35.05 kcal/mol, −21.52 kcal/mol and −22.94 kcal/mol) is more exothermic than that of R2 abstracted by OH, H and Cl (−3.40 kcal/mol, 10.13 kcal/mol and 8.55 kcal/mol), respectively. These results indicate that R1 is much easier to form and stable than R2, and R2 may be more active and short-lived. From Figure 1, DR can be formed from both R1 and R2. The potential barrier of DR from R1 abstracted by OH, H and Cl radicals is 4.12 kcal/mol, 15.91 kcal/mol and 7.48 kcal/mol, respectively, while the potential barrier of DR from R2 abstracted by OH, H and Cl radicals is 8.60 kcal/mol, 3.44 kcal/mol and −8.24 kcal/mol, respectively. Thus, it is evident that DR is more likely to form through R2 than through R1 abstracted by H and Cl radical, while the order is contrarily abstracted by OH radical.

Comparing with the values from the formation of 2-chlorinated phenoxy radical, 2-chlorinated phenyl radical and 2-chlorinated phenoxyl diradical from 2-CP with OH calculated by Pan at BB1K/6-311+G(3df,2p)//BB1K/6-311G(d,p) level [36], the potential barrier for the formation of R1 from 2,4-DCTP with OH in this study is higher by 5.30 kcal/mol than that of 2-chlorinated phenoxy radical formation from 2-CP with OH, while the potential barrier for the formation of R2 from 2,4-DCTP with OH in this study is higher by 0.39 kcal/mol than that of 2-chlorinated phenoxy radical formation from 2-CP with OH. This indicates that hydroxyl-H and phenyl-H abstraction in CP abstraction may process more easily than that of sulfydryl-H and thiophenyl-H abstraction in CTP. In addition, it is also necessary to compare our data with the value from formation of 2-chlorinated thiophenoxy radical, 2-chlorinated thiophenyl radical and 2-chlorinated thiophenoxyl diradical from 2-CTP calculated by Yu at BB1K/6-311+G(3df,2p)//BB1K/6-311G(d,p) level [30]. The formation of DR from R1 in our study can occur with a higher potential barrier by 6.55 kcal/mol and being more endothermic by 6.96 kcal/mol than those of formation of 2-chlorinated thiophenoxyl diradical from 2-chlorinated thiophenoxy radical, which implied that the diradical formation could occur with added difficulty with the substitition of Cl atom in the para-position of CTP.

### 3.2. Formation of PCTAs from R1, R2 and DR

As presented in Figure 3a, the S•/σ-C• coupling is strongly exothermic (−77.43 kcal/mol) and other three coupling modes are endothermic (8.92 kcal/mol, 44.35 kcal/mol and 37.03 kcal/mol). Thus, S•/σ-C• coupling is thermodynamically preferred to S•/σ-CCl, SH/σ-CCl and SH/σ-CH couplings, i.e., the formation of IM2 is preferred over the formation of IM1, IM3 and IM4. Only the subsequent reaction steps of IM2 to afford PCTAs are investigated in pathway 1–4. The ring closure and intra-annular elimination of Cl require the highest potential barrier (13.86 kcal/mol) and is most endoergic (18.24 kcal/mol) and is regarded as the rate-determining step for pathway 1. The ring closure and elimination of H steps occur separately, and the rate-determining step is the elimination of H for pathway 2, which has the highest potential barrier. Evidently, pathway 1 involves one less elementary steps compared to pathway 2. In addition, the rate determining step involved in pathway 1 has a lower potential barrier and is less endoergic (Δ*E* 13.86 kcal/mol, Δ*H* 18.24 kcal/mol) than that involved in pathway 2 (Δ*E* 33.22 kcal/mol, Δ*H* 27.96 kcal/mol). Therefore, pathway 1 is energetically preferred to pathway 2. Analogously, pathway 4 energetically preferred to pathway 3. Comparing pathway 1 and pathway 4, pathway 4 involves two more elementary steps (smiles rearrangement) than pathway 1. However, the rate-determining step involved in pathway 1 requires crossing a higher barrier and is more endothermic than pathway 4 (Δ*E* 12.52 kcal/mol, Δ*H* 17.09 kcal/mol). Therefore, pathway 1 and pathway 4 should be competitive. Thus, pathway 1 and pathway 4 are favored over the PCTA formation route, resulting in two dominant PCTA products (1,3,8-TCTA and 1,3,7-TCTA) from the coupling of R1 and R2.

It is evident from Figure 3b that the formation of IM5 from S•/σ-C• coupling (−78.51 kcal/mol) are much more exothermic than the formation of IM11 and IM12 from S•/σ-CCl couplings (−10.42 kcal/mol and −10.34 kcal/mol) and formation of IM13 from S•/σ-CH coupling (−11.03 kcal/mol). Thus, IM5 is preferentially easier to form than IM11, IM12 and IM13. Only one intermediate IM5 was subject to further studies for the subsequent reaction to affording PCTAs in pathways 5–8. It can be seen that pathways 5–8 are similar to pathways 1–4 and have one step less (sulfydryl-H abstraction) than pathways 1–4, respectively. Similar to cross-coupling of R1 and R2 in pathways 1–4, pathways 5 and 8 are competitive, and are the thermodynamically preferred PCTA formation route compared to pathways 6 and 7. 1,3,8-TCTA and 1,3,7-TCTA are also the main products from the coupling of R1 and DR.

Analogously, in the R2 + DR pathway of Figure 3c, the formation of IM14 from S•/σ-C• coupling (−76.28 kcal/mol) are much more exothermic than formation of IM15 from S•/σ-CCl coupling (9.53 kcal/mol), formation of IM17 from SH/σ-CCl coupling (46.16 kcal/mol) and formation of IM16 from SH/σ-C• coupling (−37.79 kcal/mol). Thus, IM14 is more easily to form than IM15, IM16 and IM17. Subsequently, after the initial sulfur-carbon coupling, IM14 evolves into 2,4,7,9-TeCTA though only one step: ring closure and elimination of H, which is the rate-determining step for pathway 9. It should be noted that the elimination of H step in pathways 2, 3, 6 and 7 is a separate reaction and causes the loss of the carbon-H, while the ring closure and elimination of H step in pathway 9 is the concerted reaction and causes the loss of the sulfydryl-H. In addition, the ring closure and elimination of H step in pathway 9 involves a much lower potential barrier (4.39 kcal/mol) and is less endoergic (2.96 kcal/mol) than the elimination of H step in pathways 2, 3, 6 and 7, which would proceed readily to yield 2,4,7,9-TeCTA at high temperature conditions.

In Figure 3d, the S•/σ-C• coupling of DR + DR can directly afford 2,4,7,9-TeCTA and release much more heats (−151.23 kcal/mol) than the S•/σ-CCl couplings to form IM18. Moreover, the two coupling modes of R2 + R2 are found to be energetically not preferred by absorbing 62.46 kcal/mol heats and releasing 21.80 kcal/mol heats, respectively, which is not competitive with the carbon-carbon coupling with a large energy release of 113.98 kcal/mol. Thus, the mainly R2 + R2 coupling product is PCDT rather than PCTA.

Comparison of the reaction pathways denoted in Figure 3a–d with previous research of R1 + R1 clearly shows that different radical couplings has a significant influence on the PCTA formation mechanism, especially on the sulfur-carbon coupling of CTPR [29]. The exothermicities of the sulfur-carbon coupling for R1 + R2, R1 + DR, R2 + DR, DR + DR, and R1 + R1 are 77.43, 78.51, 76.28, 151.23, and 11.64–11.99 kcal/mol. In addition, the potential barrier of the rate determining step for R1 + R2, R1 + DR, R2 + DR, DR + DR, and R1 + R1 is 12.52–13.86, 12.52–13.86, 4.39, 0.00 and 12.52–15.25 kcal/mol. Moreover, the number of elementary steps for R1 + R2, R1 + DR, R2 + DR, DR + DR, and R1 + R1 are 3–5, 2–4, 2, 1 and 3–5. Thus, the ranking of the PCTA formation potential is as follows: DR + DR > R2 + DR > R1 + DR > R1 + R2 > R1 + R1. It is also interesting to compare our date with the value with PCDD formation from coupling of oxygen-substituted radicals by Pan et al. at BB1K/6-311+G(3df,2p)//BB1K/6-311G(d,p) level [36].The potential barriers of the rate determining step of the oxygen-carbon coupling for 2-chlorinated R1 + R2, R1 + DR, and R1 + R1 are all 34.5 kcal/mol, which is higher than that of sulfur-carbon coupling for R1 + R2, R1 + DR and R1 + R1 in this study (13.86 kcal/mol) and our recent work [29]. In addition, the one-step reaction of oxygen-carbon coupling for 2-chlorinated R6 + R6 (−41.2 kcal/mol) in Pan’s study is less exothermic than that of sulfur-carbon coupling for R6 + R6 in this study (−151.23 kcal/mol). The result indicates that the sulfureted dioxin compounds could be produced much more readily than the corresponding oxygenated dioxin systems.

### 3.3. Formation of PCDTs from R1, R2 and DR

From Figure 4a, the formation of IM19 from σ-CCl/σ-CCl coupling and formation of IM22 from σ-CH/σ-CCl coupling have high potential barriers (47.84 kcal/mol and 44.96 kcal/mol) and are strongly endothermic (41.75 kcal/mol and 37.43 kcal/mol), while the formation of IM20 from σ-CH/σ-C• coupling and formation of IM21 from σ-CCl/σ-C• coupling are barrierless and strong exothermic (−47.28 kcal/mol and −38.93 kcal/mol). Thus, the formation of IM20 and IM21 are preferred over the formation of IM19 and IM22. Only the subsequent reaction steps of IM20 and IM21 to form PCDTs are investigated in pathway 11 and pathway 12. It can be seen that the ring closure step has the largest potential barrier and is the rate-determining step in each pathway. Notably, the rate-determining step involves in the formation of 2,6,8-TCDT in pathway12 requires a higher potential barrier (18.22 kcal/mol) than that involves in the formation of 2,4,6,8-TeCDT(16.48 kcal/mol) in pathway 11. Moreover, the formation of 2,6,8-TCDT in pathway 12 are more endothermic (5.37 kcal/mol) than the formation of 2,4,6,8-TeCDT in pathway 11 (3.45 kcal/mol). Thus, pathway 11 is expected to compete against pathway 12 for PCDT formation, and 2,4,6,8-TeCDT is the main product from the coupling of R1 and R2.

In Figure 4b, the formation of IM27 and formation of IM30 have high potential barriers (24.63 kcal/mol and 29.89 kcal/mol) and are strongly endothermic (19.25 kcal/mol and 26.90 kcal/mol), while the formation of IM28 and formation of IM29 from R1 + DR are barrierless and strong exothermic (−37.18 kcal/mol and −47.51 kcal/mol). Thus, the formation of IM28 and IM29 are overwhelmingly superior to the formation of IM27 and IM30. In addition, IM28 could not contribute to the formation of PCDT, because IM28 has no transferable H atom to form -SH, which needs to be eliminated in the PCDT formation. Thus, only the intermediate IM29 was further studied for the subsequent reaction to form 2,4,6,8-TeCDT. It can be seen that the potential barrier of the H shift step with water (14.42 kcal/mol) is higher than that of the direct H shift step without water (−2.13 kcal/mol), which indicates that water molecular has a negative catalytic role on the H shift and hinders the formation of PCDTs.

In Figure 4c, the formation of IM23 via R2 + DR releasing 111 kcal/mol heats in pathway 14 is shown. Analogously to the coupling of R1 + DR, the formation of IM32 has a high potential barrier and the formations of IM31 and IM33 hinder the formation of PCDTs. Thus, only the subsequent reactions of IM23 were studied in pathway 14, which covers only three elementary processes with one less step to form 2,4,6,8-TeCDT compared with pathway 11, 13 and 15. This means that pathway 14 is preferred over pathways 11–13. The σ-C•/σ-C• coupling to form IM34 in Figure 4d is barrierless and is more exothermic (113.98 kcal/mol) compared to the other two coupling modes to form IM35 and IM36. Following the similar elementary steps as R1 + R2 in Figure 4a, IM34 leads to the formation of 2,4,6,8-TeCDT.

Similarly, comparison of the reaction pathways presented in Figure 4a–d with previous research of R1 + R1 also indicate that different radical couplings can influence the PCDT formation [29]. The R1 + R2, R1 + DR, R2 + DR, and R2 + R2 couplings in this study are exothermic and barrierless (38.93–47.28, 47.51, 111.44, 113.98 kcal/mol), while the R1 + R1 coupling is endothermic (12.65–17.91 kcal/mol) and with high potential barriers. In addition, the rate determining step of R1 + R2, R1 + DR, R2 + DR, and R2 + R2 occurs in the ring closure step (16.48 kcal/mol) and are lower than that of R1 + R1 coupling, where the rate determining step occurs in the carbon-carbon coupling step and incurs high potential barriers (22.09–24.46 kcal/mol). Thus, the formation of PCDT from R1 + R2, R1 + DR, R2 + DR, and R2 + R2 coupling can occur more efficiently than that from R1 + R1, and the ranking of the PCDT formation potential is as follows: R2 + DR > R2 + R2 > R1 + DR > R1 + R2 > R1 + R1. Comparing with the values of rate determining step from the PCDF formation from coupling of oxygen-substituted radicals by Pan at BB1K/6-311+G(3df,2p)//BB1K/6-311G(d,p) level [36], the potential barriers for 2-chlorinated R1 + R2, R2 + DR and R2 + R2 and R1 + R1 are all 31.3 kcal/mol, which is much higher than those for R1 + R2, R2 + DR and R2 + R2 (16.48 kcal/mol) in this study and that of R1 + R1 (22.09 kcal/mol) in our previous study [29]. The result also implies that PCDF formation from sulfur substituted self- and cross- radical-radical coupling reactions can occur more readily than PCDT formation from oxygen substituted coupling reactions, which may be explained by the differences induced by the different electronic properties of S and O.

### 3.4. Rate Constant Calculations

The thermodynamic analysis of formation of R1 and R2 in Figure 1 shows that R1 is more stable and easier to form than R2. Comparision of the calculated CVT/SCT rate constants in these two steps also provided this conclusion. For example, at 1000 K, the CVT/SCT rate constant for the reaction of 2,4-DCTP + H → R1 + H_2_ via TS5 is 3.00 × 10^−12^ cm^3^ molecule^−1^ s^−1^, which is larger than the value 1.32 × 10^−14^ cm^3^ molecule^−1^ s^−1^ for the reaction of 2,4-DCTP + H → R2 + H_2_ via TS6. Analogously, comparing of the calculated CVT/SCT rate constants for the formation of DR from R1 or R2 can reflect the conclusion in the thermodynamic analysis above that DR is more likely to form through R2 abstracted by H and Cl radicals, while for the OH radical abstracting reaction, DR is more likely to form through R1. For example, at 1000 K, CVT/SCT rate constants for the reaction of R2 + H → DR + H_2_ via TS8 is 9.46 × 10^−13^ cm^3^ molecule^−1^ s^−1^, whereas the calculated value for the reaction of R1 + H → DR + H_2_ via TS7 is 1.16 × 10^−14^ cm^3^ molecule^−1^ s^−1^. In contrast, at 1000 K, the CVT/SCT rate constants are 1.56 × 10^−13^ and 8.07 × 10^−15^ cm^3^ molecule^−1^ s^−1^ for reactions R1 + OH → DR + H_2_O via TS3 and R2 + OH → DR + H_2_O via TS4.

To confirm the route possibility of the PCTA/DT formation, it is important to compare the CVT/SCT rate constants of the rate-determining step in each pathway. For example, for the formation of PCTAs in pathway 1 and pathway 2, the calculated CVT/SCT rate constants is 3.58 × 10^8^ s^−1^ for the reaction IM5 → 1,3,8-TCTA + Cl via TS17 at 1000 K, which is larger than the value 5.99 × 10^5^ s^−1^ for the IM6 → 1,3,6,8-TeCTA + H via TS19 at 1000 K. This reconfirms the finding of the thermodynamic analysis that pathways involved the elimination of Cl prefer over the pathways involved the elimination of H in the formation of PCTAs. For the formation of PCDTs in pathway 11 and pathway 12, at 1000 K, the calculated CVT/SCT rate constants of IM23 → IM24 via TS32 in 2,4,6,8-TeCDT formation pathways is 7.24 × 10^7^ s^−1^, which is larger than the value 7.07 × 10^7^ s^−1^ of IM25 → IM26 via TS38 in 2,6,8-TCDT formation pathways. This is consistent with the thermodynamic analysis: formation of 2,4,6,8-TeCDT is easier to facilitate than the formation of 2,6,8-TCDT.

## 4. Materials and Methods

Quantum chemistry calculations were carried out in the framework of density functional theory (DFT) using the Gaussian 09 program package [45]. The geometries optimizations for reactants, intermediates, transition states and products were conducted using the hybrid meta functional, MPWB1K, which gives uniformly excellent performance for thermochemistry, thermochemical kinetics, hydrogen bonding, and weak interactions, with the standard 6-31+G(d,p) basis set [46]. The vibrational frequencies were also calculated at the MPWB1K/6-31+G(d,p) level in order to determine the nature of the stationary points, the zero-point energy (ZPE), and the thermal contributions to the free energy of activation. Intrinsic reaction coordinate (IRC) calculations and the minimum energy paths (MEPs) were performed to verify that each transition state is connected to two desired minima [47]. To obtain more reliable potential barriers and reaction heats, the single-point energy calculations were refined using a more flexible basis set, 6-311+G(3df,2p), based on the optimized geometries. All the energies quoted and discussed in this paper include ZPE corrections. The kinetic constant calculations were performed using the POLYRATE 9.7 program [48]. The rate constants were calculated using canonical variational transition-state theory (CVT), and quantum tunneling corrections were considered by the small curvature tunneling (SCT) method [41,42,43,44]. The rate constants of each reaction were calculated over a wide temperature range (600–1200 K), which covers the possible formation temperature of PCTA/DTs in municipal waste incinerators [41,42,43,44].

## 5. Conclusions

In this study, mechanisms were used to investigate the homogenous gas-phase formation of PCTA/DTs from self/cross-coupling of 2,4-dichlorothiophenoxy radical (R1), 2-sulfydryl-3,5-dichlorophenyl radical (R2) and 3,5-dichlorothiophenoxyl diradical (DR). The formation reactions of R1, R2 and DR abstracted by OH, H, Cl radicals were also studied. Several energetically preferred routes for PCTA/DTs formation were proposed. The formation potential for PCTA/DT formation from different coupling were sorted and compared with our previous study. Four specific conclusions can be drawn.

(1) Besides the chlorothiophenoxy radical (R1), the sulfydryl-substituted phenyl radical (R2) and the thiophenoxyl diradical (DR) also contribute to the formation of PCTA/DTs, and the PCTA/DT formation pathways proposed in this study are proved to be both thermodynamically and kinetically feasible.

(2) The main PCTA products from self- and cross-coupling of R1, R2 and DR are 1,3,7-TCTA, 1,3,8-TCTA and 2,4,7,9-TeCTA. The S•/σ-C• condensation with both thiophenolic sulfur in one radical and *ortho* carbon in the other radical bonded to single electron is the most efficient sulfur-carbon coupling mode, and the order of the PCTA formation potential is as follows: DR + DR > R2 + DR > R1 + DR > R1 + R2 > R1 + R1.

(3) The main PCDT products from self- and cross-coupling of R1, R2 and DR are 2,4,6,8-TeCDT. The σ-C•/σ-C• coupling with both *ortho* carbon in the two radicals bonded to single electron is the energetically favored carbon-carbon coupling mode, and the ranking of the PCDT formation potential is: R2 + DR > R2 + R2 > R1 + DR > R1 + R2 > R1 + R1.

(4) The PCTA/DTs produced from R1, R2 and DR can occur much more readily than PCDD/DFs formation from corresponding oxygen substituted radicals.

## Figures and Tables

**Table 1:** Arrhenius formulas for crucial elementary reactions involved in the formation of R1, R2 and DR from the 2,4-DCTP precursor and PCTA/DTs from the R1, R2 and DR precursors over the temperature range of 600–1200 K. (units are s^−1^ and cm^3^ molecule^−1^ s^−1^ for unimolecular and bimolecular reactions, respectively. ^a^ Reproduced with permission from Xu et al. [39], ^b^ Reproduced with permission from Xu et al. [29]).

Reactions Arrhenius Formulas	Arrhenius Formulas
2,4-DCTP + OH → R1 + H_2_O via TS1 ^a^	*k*(T) = (6.69 × 10^−14^) exp (−5861/T)
2,4-DCTP + OH → R2 + H_2_O via TS2	*k*(T) = (1.07 × 10^−15^) exp (−12857/T)
R1 + OH → DR + H_2_O via TS3	*k*(T) = (7.18 × 10^−12^) exp (−3795/T)
R2 + OH → DR + H_2_O via TS4	*k*(T) = (3.19 × 10^−13^) exp (−3630/T)
2,4-DCTP + H → R1 + H_2_ via TS5 ^a^	*k*(T) = (2.84 × 10^−11^) exp (−2229/T)
2,4-DCTP + H → R2 + H_2_ via TS6	*k*(T) = (9.24 × 10^−11^) exp (−8977/T)
R1 + H → DR + H_2_ via TS7	*k*(T) = (6.55 × 10^−11^) exp (−8600/T)
R2 + H → DR + H_2_ via TS8	*k*(T) = (3.99 × 10^−12^) exp (−1331/T)
2,4-DCTP + Cl → R2 + HCl via TS10	*k*(T) = (1.52 × 10^−10^) exp (−5852/T)
R1 + Cl → DR + HCl via TS11	*k*(T) = (1.47 × 10^−10^) exp (−5978/T)
IM2 + H → IM5 + H_2_ via TS13	*k*(T) = (3.53 × 10^−11^) exp (−2623/T)
IM2 + SH → IM5 + H_2_S via TS15	*k*(T) = (8.24 × 10^−13^) exp (−3080/T)
IM5 → 1,3,8-TCTA + Cl via TS17 ^b^	*k*(T) = (4.52 × 10^11^) exp (−7140/T)
IM5 → IM6 via TS18 ^b^	*k*(T) = (6.15 × 10^11^) exp (−4332/T)
IM6 → 1,3,6,8-TeCTA + H via TS19 ^b^	*k*(T) = (2.84 × 10^13^) exp (−17667/T)
IM14 → 2,4,7,9-TeCTA + H via TS26	*k*(T) = (4.52 × 10^11^) exp (−2395/T)
IM20 + H → IM23 + H_2_ via TS29	*k*(T) = (1.00 × 10^−11^) exp (−1531/T)
IM23 → IM24 via TS32 ^b^	*k*(T) = (3.07 × 10^11^) exp (−8241/T)
IM24 → 2,4,6,8-TeCDT + SH via TS33 ^b^	*k*(T) = (5.86 × 10^12^) exp (−351/T)
IM21 + H → IM25 + HCl via TS34	*k*(T) = (9.78 × 10^−11^) exp (−2502/T)
IM21 + OH → IM25 + HOCl via TS35	*k*(T) = (1.13 × 10^−11^) exp (−12954/T)
IM21 + SH → IM25 + HSCl via TS36	*k*(T) = (4.82 × 10^−12^) exp (−3861/T)
IM25 → IM26 via TS38	*k*(T) = (7.61 × 10^11^) exp (−9275/T)
IM26 → 2,6,8-TCDT + SH via TS39	*k*(T) = (8.96 × 10^12^) exp (−275/T)
IM29 + H_2_O → IM23 + H_2_O via TS43 ^b^	*k*(T) = (3.20 × 10^−15^) exp (−3310/T)
IM34 + H → IM23 + H_2_ via TS46 ^b^	*k*(T) = (5.71 × 10^−9^) exp (−1148/T)
IM34 + OH → IM23 + H_2_O via TS47 ^b^	*k*(T) = (7.66 × 10^−12^) exp (−1889/T)
IM34 + SH → IM23 + H_2_S via TS48 ^b^	*k*(T) = (1.10 × 10^−12^) exp (−2808/T)

## Supplementary Materials

Supplementary Materials can be found at https://www.mdpi.com/1422-0067/20/11/2623/s1. Figure S1. PCTA/DTs formation routes embedded with the reaction heats Δ*H* (in kcal/mol) from self-couplings of R2 and DR. Δ*H* is calculated at 0 K. Table S1. The potential barriers Δ*E* (in kcal/mol) and reaction heats Δ*H* (in kcal/mol) of several typical reactions in the formation of PCTA/DTs at MPWB1K/6-311+G(3df,2p)//MPWB1K/6-31+G(d,p) and BB1K/6-311+G(3df,2p) //BB1K/6-311G(d,p) levels. Table S2. Imaginary frequencies (in cm^−1^), zero point energies (ZPE, in a.u.) and total energies (in a.u.) for the transition states involved in the formation of PCTA/DTs from R1, R2 and DR. Table S3. CVT/SCT rate constants for the formation of PCTA/DTs from R1, R2 and DR over the temperature range of 600−1200 K (units are s^−1^ and cm^3^ molecule^−1^ s^−1^ for unimolecular and bimolecular reactions, respectively). Table S4. Cartesian coordinates for the transition states involved in the formation of PCTA/DTs from R1, R2 and DR. Table S5. Cartesian coordinates for the reactants, intermediates and products involved in the formation of PCTA/DTs from R1, R2 and DR.

## Abbreviations

PCDTs	polychlorinated dibenzothiophenes
PCTAs	polychlorinated thianthrenes
PCDDs	polychlorinated dibenzo-p-dioxins
PCDFs	polychlorinated dibenzofurans
R1	2,4-dichlorothiophenoxy radical
R2	2-sulfydryl-3,5-dichlorophenyl radical
DR	3,5-dichlorothiophenoxyl diradical
1,3,8-TCTA	1,3,8-trichlorothianthrene
1,3,6,8-TeCTA	1,3,6,8-tetrachlorothianthrene
1,3,7,9-TeCTA	1,3,7,9-tetrachlorothianthrene
1,3,7-TCTA	1,3,7-trichlorothianthrene
2,4,7,9-TeCTA	2,4,7,9-tetrachlorothianthrene
2,4,6,8-TeCDT	2,4,6,8-tetrachlorodibenzothiophene
2,6,8-TeCDT	2,6,8-trichlorodibenzothiophene
DFT	density functional theory
TST	transition state theory
IRC	intrinsic reaction coordinate
ZPE	zero-point energy
MEPs	minimum energy paths
CVT	canonical variational transition-state theory
SCT	small curvature tunneling
