# Supplementary material for: Mechanistic and Kinetic Study on Self-/Cross- Condensation of PCTA/DT Formation Mechanisms from Three Types of Radicals of 2,4-Dichlorothiophenol
Source: Int J Mol Sci. 2019 May 28;20(11):2623. doi: 10.3390/ijms20112623 (PMC6600164; doi:10.3390/ijms20112623)
Supplement: Supplementary file 1 [file ijms-20-02623-s001.pdf]

Supplementary Information for

Mechanistic and Kinetic Study on Self-/Cross-Condensation of PCTA/DT Formation Mechanisms from Three Types of Radicals of 2,4-Dichlorothiophenol

Hetong Wang [1], Chenpeng Zuo [1], Siyuan Zheng [1], Yanhui Sun [2], Fei Xu [1,3,*] and Qingzhu Zhang [1]

[1] Environment Research Institute, Shandong University, Qingdao 266237, China; Kishi_Wang@163.com (H.W.); zuochenpeng@126.com (C.Z.); zhengsiyuan1991@126.com (S.Z.); zqz@sdu.edu.cn (Q.Z.)

[2] College of Environment and Safety Engineering, Qingdao University of Science & Technology, Qingdao 266042, China; sunyh0532@126.com

[3] Shenzhen Research Institute, Shandong University, Shenzhen 518057, China

* Correspondence: xufei@sdu.edu.cn; Tel.: + 86-532-5863-1992

Contains one figure and five tables

Figure S1. PCTA/DTs formation routes embedded with the reaction heats ΔH (in kcal/mol) from self-couplings of R2 and DR. ΔH is calculated at 0 K.

Table S1. The potential barriers ΔE (in kcal/mol) and reaction heats ΔH (in kcal/mol) of several typical reactions in the formation of PCTA/DTs at MPWB1K/6-311+G(3df,2p)//MPWB1K/6-31+G(d,p) and BB1K/6-311+G(3df,2p) //BB1K/6-311G(d,p) levels.

Table S2. Imaginary frequencies (in cm^{-1}), zero point energies (ZPE, in a.u.) and total energies (in a.u.) for the transition states involved in the formation of PCTA/DTs from R1, R2 and DR.

Table S3. CVT/SCT rate constants for the formation of PCTA/DTs from R1, R2 and DR over the temperature range of 600−1200 K (units are s^{-1} and cm^3 $molecule^{-1}$ s^{-1} for unimolecular and bimolecular reactions, respectively).

Table S4. Cartesian coordinates for the transition states involved in the formation of PCTA/DTs from R1, R2 and DR.

Table S5. Cartesian coordinates for the reactants, intermediates and products involved in the formation of PCTA/DTs from R1, R2 and DR.

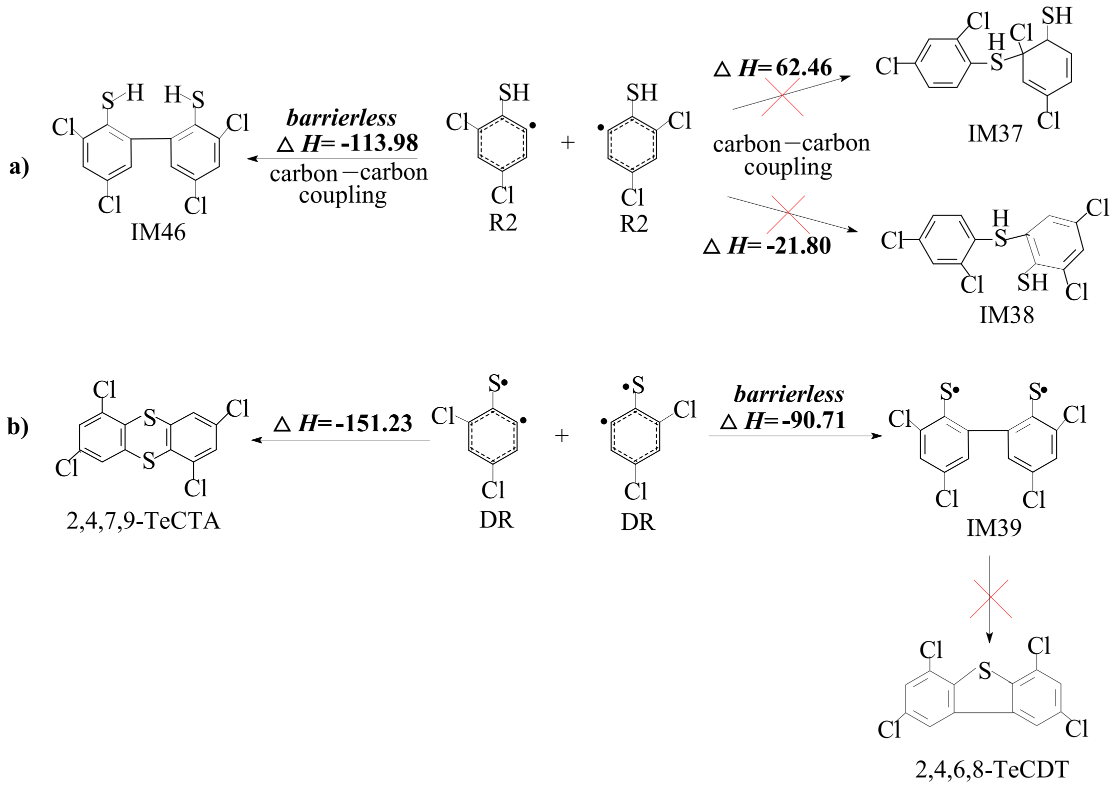

Figure S1. PCTA/DTs formation routes embedded with the reaction heats Δ*H* (in kcal/mol) from self-couplings of R2 and DR. Δ*H* is calculated at 0 K.

Table S1. The potential barriers ΔE (in kcal/mol) and reaction heats ΔH (in kcal/mol) of several typical reactions in the formation of PCTA/DTs at MPWB1K/6-311+G(3df,2p)//MPWB1K/6-31+G(d,p) and BB1K/6-311+G(3df,2p) //BB1K/6-311G(d,p) levels.

Reactions	MPWB1K		BB1K	
	ΔE	ΔH	ΔE	ΔH
2,4-DCTP + OH → R1 + H_2O via TS1	8.81	−35.27	8.37	−35.27
R1 + OH → DR + H_2O via TS3	4.12	−4.44	4.34	−4.61
2,4-DCTP + H → R1 + H_2 via TS5	3.45	−21.51	3.19	−21.78
R1 + H → DR + H_2 via TS7	15.91	9.09	15.43	8.89
IM2 + H → IM5 + H_2 via TS13	3.63	−19.67	3.37	−19.87
IM5 → 1,3,8-TCTA + Cl via TS17	13.86	18.24	13.98	17.47
IM5 → IM6 via TS18	8.07	3.75	8.09	4.01
IM6→ 1,3,6,8-TeCTA + H via TS19	33.22	27.96	33.16	29.15
IM20 + H → IM23 + H_2 via TS29	1.47	−55.07	1.51	−54.96
IM23 → IM24 via TS32	16.48	3.45	16.35	3.58
IM24 → 2,4,6,8-TeCDT + SH via TS33	0.09	−9.97	−0.02	−11.09

Table S2. Imaginary frequencies (in cm^{-1}), zero point energies (ZPE, in a.u.) and total energies (in a.u.) for the transition states involved in the formation of PCTA/DTs from R1, R2 and DR.

Transition states	Imaginary frequencies	ZPE	Total energies
TS1	2518i	0.091923	−1625.52895891
TS2	1763i	0.088167	−1625.52953405
TS3	1796i	0.078620	−1624.89916655
TS4	2507i	0.078502	−1624.84149242
TS5	889i	0.082758	−1550.30695718
TS6	973i	0.080418	−1550.28397541
TS7	1007i	0.071210	−1549.65160160
TS8	868i	0.069532	−1549.61936749
TS9	837i	0.080849	−2010.03652902
TS10	168i	0.076286	−2010.00586551
TS11	381i	0.066234	−2009.37294733
TS12	838i	0.067642	−2009.34899380
TS13	954i	0.146744	−3098.92758591
TS14	906i	0.156527	−3174.16498923
TS15	1111i	0.151518	−3497.23296726
TS16	892i	0.145213	−3558.65585021
TS17	418i	0.136723	−3097.77653953
TS18	343i	0.136999	−3097.78604123
TS19	1069i	0.129826	−3097.73281895
TS20	398i	0.136854	−3097.78500123
TS21	398i	0.136636	−3097.78383493
TS22	331i	0.136983	−3097.78581033
TS23	1074i	0.129858	−3097.73233331
TS24	392i	0.136760	−3097.78492886
TS25	411i	0.136764	−3097.77690142
TS26	588i	0.129869	−3097.73678571
TS27	425i	0.143785	−3098.23296529
TS28	392i	0.143949	−3098.23772483
TS29	1010i	0.145884	−3098.88213192
TS30	1222i	0.156639	−3174.12226690
TS31	1369i	0.151459	−3497.19591903
TS32	361i	0.134647	−3097.77868237
TS33	424i	0.134393	−3097.79903436
TS34	813i	0.147702	−3098.86766669
TS35	512i	0.157534	−3174.06867348
TS36	416i	0.154981	−3497.17237420
TS37	282i	0.146520	−3558.58659699
TS38	363i	0.144308	−2638.09827302

Table S2. *Cont*.

Transition states	Imaginary frequencies	ZPE	Total energies
TS39	424i	0.144394	−2638.11873278
TS40	629i	0.134491	−3097.63835527
TS41	517i	0.134565	−3097.63003926
TS42	1279i	0.133629	−3097.75585590
TS43	1276i	0.158116	−3174.15297659
TS44	429i	0.130539	−3097.54457948
TS45	456i	0.139640	−3098.14299003
TS46	927i	0.144877	−3098.93273985
TS47	865i	0.154882	−3174.17011495
TS48	1034i	0.149306	−3497.23877027
TS49	877i	0.143216	−3558.66232492

Table S3. CVT/SCT rate constants for the formation of PCTA/DTs from R1, R2 and DR over the temperature range of 600−1200 K (units are s^{-1} and cm^3 $molecule^{-1}$ s^{-1} for unimolecular and bimolecular reactions, respectively).

T(K)	CVT/SCT Rate Constants			
	TS1	TS2	TS3	TS4
600	4.34×10^{-18}	5.80×10^{-25}	1.43×10^{-14}	8.68×10^{-16}
700	1.56×10^{-17}	1.09×10^{-23}	3.05×10^{-14}	1.68×10^{-15}
800	4.26×10^{-17}	1.04×10^{-22}	5.71×10^{-14}	3.02×10^{-15}
900	9.68×10^{-17}	6.24×10^{-22}	9.75×10^{-14}	5.07×10^{-15}
1000	1.93×10^{-16}	2.71×10^{-21}	1.56×10^{-13}	8.07×10^{-15}
1100	3.46×10^{-16}	9.23×10^{-21}	2.35×10^{-13}	1.23×10^{-14}
1200	5.77×10^{-16}	2.26×10^{-20}	3.40×10^{-13}	1.79×10^{-14}
	TS5	TS6	TS7	TS8
600	7.33×10^{-13}	2.89×10^{-17}	4.14×10^{-17}	3.99×10^{-13}
700	1.15×10^{-13}	2.43×10^{-16}	3.29×10^{-16}	6.45×10^{-13}
800	1.67×10^{-13}	1.24×10^{-15}	1.20×10^{-15}	9.56×10^{-13}
900	2.29×10^{-12}	4.56×10^{-15}	4.17×10^{-15}	7.37×10^{-13}
1000	3.00×10^{-12}	1.32×10^{-14}	1.16×10^{-14}	9.46×10^{-13}
1100	3.81×10^{-12}	2.38×10^{-14}	2.74×10^{-14}	1.18×10^{-12}
1200	4.71×10^{-12}	5.03×10^{-14}	5.71×10^{-14}	1.44×10^{-12}
	TS10	TS11	TS13	TS15
600	9.51×10^{-15}	7.51×10^{-15}	4.77×10^{-13}	5.31×10^{-15}
700	3.46×10^{-14}	2.79×10^{-14}	8.11×10^{-13}	9.78×10^{-15}
800	9.52×10^{-14}	7.82×10^{-14}	1.26×10^{-12}	1.63×10^{-14}
900	2.16×10^{-13}	1.81×10^{-13}	1.82×10^{-12}	2.51×10^{-14}
1000	4.27×10^{-13}	3.63×10^{-13}	2.50×10^{-12}	3.67×10^{-14}
1100	7.61×10^{-13}	6.57×10^{-13}	3.32×10^{-12}	5.14×10^{-14}
1200	1.25×10^{-12}	1.10×10^{-12}	4.26×10^{-12}	6.96×10^{-14}
	TS17	TS18	TS19	TS26
600	3.09×10^{6}	4.52×10^{8}	4.77	8.55×10^{9}
700	1.68×10^{7}	1.26×10^{9}	3.07×10^{2}	1.46×10^{10}
800	5.98×10^{7}	2.73×10^{9}	7.12×10^{3}	2.22×10^{10}
900	1.61×10^{8}	4.98×10^{9}	8.31×10^{4}	3.11×10^{10}
1000	3.58×10^{8}	8.08×10^{9}	5.99×10^{5}	4.10×10^{10}
1100	6.88×10^{8}	1.20×10^{10}	3.04×10^{6}	5.16×10^{10}
1200	1.19×10^{9}	1.67×10^{10}	1.18×10^{7}	6.29×10^{10}
	TS29	TS32	TS33	TS34
600	8.33×10^{-13}	2.92×10^{5}	3.27×10^{12}	1.78×10^{-12}
700	1.10×10^{-12}	2.64×10^{6}	3.55×10^{12}	2.44×10^{-12}
800	1.41×10^{-12}	1.39×10^{7}	3.78×10^{12}	3.84×10^{-12}
900	1.75×10^{-12}	2.58×10^{7}	3.97×10^{12}	5.66×10^{-12}
1000	2.12×10^{-12}	7.24×10^{7}	4.13×10^{12}	7.88×10^{-12}
1100	2.54×10^{-12}	1.69×10^{8}	4.26×10^{12}	1.05×10^{-11}
1200	3.00×10^{-12}	3.42×10^{8}	4.38×10^{12}	1.36×10^{-11}

Table S2. *Cont*.

T(K)	CVT/SCT Rate Constants			
	TS35	TS36	TS38	TS39
600	5.13×10^{-21}	8.39×10^{-15}	1.49×10^{5}	1.42×10^{13}
700	1.01×10^{-19}	1.88×10^{-14}	1.43×10^{6}	1.33×10^{13}
800	9.80×10^{-19}	3.61×10^{-14}	6.47×10^{6}	1.26×10^{13}
900	5.97×10^{-18}	6.21×10^{-14}	2.44×10^{7}	1.22×10^{13}
1000	2.60×10^{-17}	9.87×10^{-14}	7.07×10^{7}	1.18×10^{13}
1100	8.88×10^{-17}	1.48×10^{-13}	1.69×10^{8}	1.15×10^{13}
1200	2.52×10^{-16}	2.10×10^{-13}	3.50×10^{8}	1.13×10^{13}
	TS43	TS46	TS47	TS48
600	1.45×10^{-17}	9.15×10^{-10}	3.58×10^{-13}	1.11×10^{-14}
700	2.71×10^{-17}	1.07×10^{-9}	4.99×10^{-13}	1.92×10^{-14}
800	4.60×10^{-17}	1.27×10^{-9}	6.72×10^{-13}	3.04×10^{-14}
900	7.32×10^{-17}	1.50×10^{-9}	8.80×10^{-13}	4.53×10^{-14}
1000	1.11×10^{-16}	1.76×10^{-9}	1.12×10^{-12}	6.41×10^{-14}
1100	1.63×10^{-16}	2.06×10^{-9}	1.41×10^{-12}	8.74×10^{-14}
1200	2.33×10^{-16}	2.39×10^{-9}	1.74×10^{-12}	1.16×10^{-13}

Table S4. Cartesian coordinates for the transition states involved in the formation of PCTA/DTs from R1, R2 and DR.

TS1

C	2.15132900	0.06709400	−0.00012000
C	1.81242600	1.41117500	−0.00009100
C	0.48479100	1.76410400	−0.00001500
C	−0.54228500	0.81141500	0.00003700
C	−0.15854500	−0.53238700	−0.00000100
C	1.17328500	−0.90559900	−0.00007600
H	−3.04524300	0.30109800	0.00018900
H	2.58269200	2.16443800	−0.00012700
S	−2.15916100	1.43384900	0.00015400
O	−4.27178000	−0.40744300	0.00024100
H	−4.19075300	−1.36717500	0.00011800
H	0.21275500	2.80807100	0.00001000
Cl	−1.32549600	−1.79609000	0.00004600
H	1.43779300	−1.94962400	−0.00010200
Cl	3.80770600	−0.40000800	−0.00021500

TS2

C	0.24418300	−1.04834000	0.00310600
C	0.97403000	0.13966500	−0.02409600
C	0.22980400	1.30255500	−0.04383700
C	−1.13961600	1.35972300	−0.04305000
C	−1.82271900	0.15478600	−0.00991400
C	−1.13960300	−1.04500700	0.02074300
H	0.82890300	2.41059700	−0.06543300
H	−1.66531300	2.30014900	−0.05552500
H	−1.67611800	−1.97824700	0.05074000
Cl	1.04839500	−2.57209300	0.03092300
O	1.50366500	3.39030500	0.06707400
H	2.25121400	3.03004600	0.55821000
Cl	−3.54472900	0.14714900	0.00134000
S	2.72578600	0.25149900	−0.09852700
H	2.98063300	−0.86521300	0.58567900

TS3

C	0.26350500	−1.06449800	−0.00014400
C	1.02425800	0.13477100	0.00013200
C	0.26999000	1.31556300	0.00009200
C	−1.09339300	1.38387400	0.00020600

C	−1.78282300	0.17616300	0.00009100
C	−1.11381400	−1.04011700	−0.00014100
H	0.87936900	2.41974400	−0.00026900
H	−1.61862100	2.32463500	0.00038700
H	−1.66971400	−1.96233600	−0.00031200
Cl	1.04320700	−2.58657500	−0.00049900
O	1.59499600	3.36963200	−0.00108300
H	2.44696800	2.91491200	−0.00032000
Cl	−3.49665300	0.18132500	0.00018700
S	2.71901800	0.17504400	0.00081700

TS4

C	2.15940500	0.10966700	−0.00011500
C	1.82336300	1.46084600	−0.00007500
C	0.49162000	1.72154600	0.00000200
C	−0.56336300	0.83339500	0.00004500
C	−0.15803500	−0.51096600	0.00000200
C	1.17968100	−0.86653100	−0.00007700
H	−3.04870600	0.33962100	0.00016500
H	2.57776800	2.23052800	−0.00010400
S	−2.16990900	1.47723300	0.00014300
O	−4.27181500	−0.38063200	0.00021500
H	−4.18298100	−1.33987400	0.00019600
Cl	−1.31511500	−1.78401200	0.00004600
H	1.45333500	−1.90786800	−0.00010900
Cl	3.81497500	−0.35721300	−0.00021400

TS5

C	−0.85444300	0.89367200	−0.00463700
C	0.49903600	0.61032200	−0.03239600
C	0.96488100	−0.70313900	−0.02050100
C	0.02111300	−1.72466700	0.00819900
C	−1.33375700	−1.46202600	0.00995600
C	−1.76084400	−0.14752800	0.00898000
H	0.36766600	−2.74563100	0.03442300
H	−2.05042700	−2.26627500	0.02709800
H	3.10857600	−0.31979900	0.91695000
H	3.41594900	0.36994500	1.96345500
Cl	1.59550800	1.93672000	−0.07371200
S	2.66418000	−1.13481600	−0.12611900
H	−1.19263600	1.91614100	−0.00454100
Cl	−3.44797400	0.20462500	0.03035300

TS6

C	−1.73541900	0.13676800	−0.00020100
C	−0.84440600	−0.91724800	0.00142800
C	0.51937600	−0.67147900	0.00005100
C	1.02821100	0.62778400	0.00168900
C	0.07492900	1.62263800	0.00088500
C	−1.27867700	1.44681800	−0.00082500
H	−1.20194600	−1.93298900	0.00207500
H	0.55376200	3.01998700	0.01111700
H	−1.96472400	2.27867200	−0.00104000
S	2.72167300	1.08161600	−0.00623300
H	3.20415400	−0.15866000	0.06905000
Cl	1.57404300	−2.04000000	−0.00054300
H	0.82279800	3.81633000	0.01874000
Cl	−3.42962400	−0.18358200	−0.00053900

TS7

C	−1.69282300	0.12608100	−0.00000600
C	−0.78698600	−0.92686400	−0.00003600
C	0.56698800	−0.66875900	−0.00001900
C	1.06792800	0.66108000	0.00003000
C	0.08211100	1.64901700	0.00005700
C	−1.26619600	1.44926500	0.00004200
H	−1.14254800	−1.94322200	−0.00007400
H	0.52928500	3.04729000	0.00010500
H	−1.97253400	2.26395500	0.00006500
S	2.71788700	1.05489300	0.00005300
Cl	1.64029700	−2.00237200	−0.00005800
H	0.75254500	3.85822000	0.00012400
Cl	−3.37436000	−0.22371400	−0.00002900

TS8

C	−0.84969800	0.85834100	−0.00825100
C	0.50718800	0.58455100	−0.04099400
C	0.98559700	−0.73066800	−0.03016100
C	0.00434100	−1.68477500	−0.00352300
C	−1.34597000	−1.50624600	0.01273900
C	−1.76293800	−0.18107700	0.01185900
H	−2.05158400	−2.32059100	0.02993400
H	3.11442000	−0.36757400	0.91747100
H	3.42553400	0.32677000	1.97380100

Cl	1.60406700	1.90995200	−0.07470800
S	2.67552900	−1.19381000	−0.11738300
H	−1.19036100	1.87963700	−0.00738400
Cl	−3.44745500	0.18075200	0.03437200

TS9

C	−0.12513900	0.67966600	−0.35450300
C	0.35685100	−0.62216900	−0.54106300
C	−1.85073000	−1.47868400	−0.04886000
C	−2.29567100	−0.17980700	0.13884300
C	−1.44300500	0.89926800	−0.00713400
H	−2.52768800	−2.30717600	0.07531400
H	−1.80121800	1.90406400	0.14036900
Cl	0.89932500	2.03754700	−0.55311200
H	2.66811000	−0.04593900	−0.23976600
Cl	−3.93519300	0.10355400	0.56198300
S	1.97609000	−0.99386200	−1.02629300
Cl	3.36190100	−0.17790000	1.40844100
C	−0.53175100	−1.68683700	−0.37810900
H	−0.16253600	−2.69221600	−0.50458900

TS10

C	1.67222100	−0.99456900	−0.00050500
C	1.82285500	0.37786900	0.00315000
C	0.71131900	1.20414900	−0.00005500
C	−0.59046400	0.69050400	−0.00035600
C	−0.62824600	−0.68229100	−0.00219900
C	0.40546500	−1.56652900	−0.00390400
H	2.80713400	0.81447700	0.00593000
H	−2.07790700	−1.39704300	0.00594500
H	0.26260000	−2.63478900	−0.00590200
Cl	0.98380800	2.90676000	0.00147000
Cl	3.06470900	−2.00523900	−0.00050500
Cl	−3.25901900	−2.04774900	0.00469800
S	−2.07966400	1.60794900	−0.01199600
H	−1.49755600	2.80124700	0.11289700

TS11

C	−1.46424700	−1.15731400	0.00001200
C	−1.82292400	0.18519000	0.00004600
C	−0.85102300	1.16263800	0.00003100
C	0.53110500	0.82971900	−0.00002600

C	0.77092500	−0.53745500	−0.00006800
C	−0.12888400	−1.55589700	−0.00004300
H	−2.86351100	0.46210200	0.00008800
H	2.22686800	−1.02381700	−0.00017000
H	0.15533300	−2.59524100	−0.00004900
S	1.80536400	1.94758000	−0.00003500
Cl	−1.33747100	2.79984400	0.00008100
Cl	−2.69625000	−2.34734500	0.00004400
Cl	3.40935500	−1.72106400	−0.00006800

TS12

C	0.12526300	−0.65645600	−0.35758100
C	−0.36719100	0.64828000	−0.54936300
C	0.56302500	1.64629100	−0.38442300
C	1.87620300	1.52167900	−0.05757400
C	2.30809600	0.21281700	0.13335300
C	1.44561200	−0.86474600	−0.01132100
H	2.54404200	2.35959200	0.05546700
H	1.80636900	−1.86784500	0.13962700
Cl	−0.90197100	−2.01222500	−0.54753900
H	−2.67032300	0.08073900	−0.26571400
Cl	3.94555700	−0.08365900	0.55404400
S	−1.97984700	1.06061600	−1.01203000
Cl	−3.37938400	0.17885300	1.38317500

TS13

C	1.43200900	−0.78357500	0.00286000
C	2.71898200	−0.91908600	−0.50839700
C	3.78700100	−0.23178500	0.03673900
C	3.56992400	0.60854300	1.10978100
C	2.30474300	0.75684000	1.64580400
C	1.25130500	0.05518900	1.09611000
Cl	3.02016700	−1.96880600	−1.84059200
H	4.77330300	−0.35235500	−0.37885000
H	0.26793500	0.16280400	1.52665600
C	−1.29882800	−0.78830900	−0.25172900
C	−2.25194400	−1.44914000	0.50672800
C	−3.44177500	−0.81903700	0.81454600
C	−3.67631500	0.47744100	0.41032200
C	−2.70694200	1.14017600	−0.31996400
C	−1.51272900	0.52560200	−0.69975200
H	−4.59390200	0.97854200	0.66821300
H	−1.16228800	1.92411300	−2.56351900

Cl	−3.04408300	2.77029200	−0.76354100
H	2.14578100	1.40860100	2.48893000
H	−2.06111100	−2.45259600	0.84942500
S	0.12342000	−1.72016300	−0.71819700
S	−0.28119600	1.34234000	−1.64217100
Cl	4.89729900	1.47535900	1.78686100
Cl	−4.63540600	−1.65039300	1.73527800
H	−1.94350100	2.28920800	−3.48937400

TS14

C	−1.52305600	−0.74766000	0.43191600
C	−2.77960800	−0.43653700	0.94084500
C	−3.85146800	−0.18108200	0.10596600
C	−3.66836500	−0.23465400	−1.26069300
C	−2.43371900	−0.55064700	−1.79634500
C	−1.37717900	−0.81301600	−0.94870100
Cl	−3.04128100	−0.36933700	2.64230400
H	−4.81401900	0.06082700	0.52424300
H	−0.41706900	−1.06984900	−1.36867200
C	1.20967100	−0.68106200	0.57477800
C	2.13303900	−1.69032600	0.35754600
C	3.31104100	−1.40894800	−0.30667500
C	3.56280000	−0.14312600	−0.79121500
C	2.62483100	0.85403200	−0.59488700
C	1.44464700	0.62487000	0.11539300
H	4.47220500	0.07161900	−1.32639600
H	1.15028500	2.91664100	0.58916700
Cl	2.97895500	2.40198400	−1.25073300
H	−2.30196500	−0.59785600	−2.86469800
H	1.92878700	−2.68821300	0.70856200
S	−0.20188000	−1.09460600	1.54845300
S	0.25266600	1.87350100	0.39434600
Cl	−4.99893000	0.09355800	−2.30572200
Cl	4.46798900	−2.65848300	−0.55605600
O	1.97899600	3.39643200	1.76303000
H	1.50696900	3.17070400	2.57471200

TS15

C	−1.63584600	−0.69265600	0.69985100
C	−2.82371300	−0.05227700	1.03672200
C	−3.89850800	−0.03117100	0.16711700

C	−3.78658300	−0.65535500	−1.05872000
C	−2.62218600	−1.30979700	−1.41680000
C	−1.56407900	−1.33212800	−0.53244900
Cl	−2.99216000	0.74103300	2.55509400
H	−4.80672400	0.47619700	0.44579900
H	−0.65806700	−1.85142000	−0.80442100
C	1.09321400	−0.71520200	0.75456300
C	1.99571200	−1.75473700	0.89543900
C	3.15132800	−1.76593000	0.13568200
C	3.39994300	−0.77727100	−0.79300500
C	2.48029400	0.24292900	−0.94841000
C	1.32512500	0.32511200	−0.16438500
H	4.29064100	−0.79961100	−1.39753100
H	1.16286400	2.73531100	−0.19119400
Cl	2.82765700	1.43520300	−2.13472800
H	1.50934800	3.86315400	1.64588800
H	−2.54835800	−1.80212900	−2.37233400
H	1.79468000	−2.55067400	1.59321000
S	−0.29294700	−0.71517100	1.84487100
S	0.18516100	1.63030800	−0.33701000
Cl	−5.11925300	−0.61833700	−2.15026900
Cl	4.28117100	−3.04727000	0.32999300
S	2.20849600	3.85732400	0.50622900

TS16

C	−1.61434300	−0.69987100	0.71541900
C	−2.80426100	−0.05142600	1.02938300
C	−3.87368000	−0.05591200	0.15211900
C	−3.75416300	−0.71424000	−1.05484300
C	−2.58738000	−1.37859000	−1.38771800
C	−1.53468500	−1.37542300	−0.49694300
Cl	−2.98156200	0.78300600	2.52263600
H	−4.78398500	0.45819400	0.41094300
H	−0.62769000	−1.90362500	−0.74793700
C	1.10919700	−0.68292200	0.77949700
C	2.02372500	−1.70715100	0.92707100
C	3.17357700	−1.71831200	0.15454200
C	3.40004700	−0.74722200	−0.79927500
C	2.47513100	0.26574900	−0.96037600
C	1.33285400	0.35205300	−0.15247700
H	4.28060500	−0.77767300	−1.41807900
H	1.13406500	2.69671600	−0.53906600
Cl	2.78473100	1.42288400	−2.18541900

H	−2.50886200	−1.89941200	−2.32766200
H	1.83892300	−2.49438600	1.63900300
S	−0.27671700	−0.68615300	1.86957400
S	0.20232300	1.65549400	−0.27475700
Cl	−5.07928600	−0.70960500	−2.15472100
Cl	4.31582400	−2.98173200	0.35964200
Cl	2.00624200	3.80840800	0.51829400

TS17

C	−1.42194600	−1.28992800	−0.33854800
C	−1.57054600	−0.22358300	0.60567100
C	−2.87175200	0.33927000	0.78325000
C	−3.89546100	−0.04594400	−0.02383200
C	−3.73110400	−1.05556600	−0.98462400
C	−2.50102800	−1.65587100	−1.12948900
Cl	−0.61860800	−0.27731400	2.06449800
H	−3.00770700	1.08685100	1.54614500
H	−2.35869600	−2.41240200	−1.88517000
C	1.31273800	−0.84772900	−0.34750800
C	2.58346900	−1.34680100	−0.09595700
C	3.64729000	−0.48095300	0.01918500
C	3.46481100	0.88450500	−0.07954300
C	2.19074900	1.36798100	−0.28855800
C	1.07971400	0.53001200	−0.43338600
H	4.29409800	1.56409900	0.01745500
Cl	1.98810700	3.07449500	−0.39501500
H	−4.56336000	−1.34629400	−1.60346500
H	2.73980300	−2.40899000	0.00380800
S	0.09667400	−2.09109900	−0.59698200
S	−0.49535800	1.22704300	−0.67869600
Cl	5.22799000	−1.10565900	0.30245400
Cl	−5.44729900	0.67843400	0.15818700

TS18

C	1.36595300	−0.85173600	0.13236800
C	2.63303900	−1.26485700	−0.24750300
C	3.74469100	−0.47645200	−0.02325900
C	3.59451500	0.75777700	0.61792200
C	2.37116400	1.20085900	1.02268900
C	1.20853700	0.44005300	0.71774500
Cl	2.84307100	−2.79370400	−1.00942000
H	4.71867400	−0.81612300	−0.33219400
H	0.35665000	0.57916300	1.36919000

C	−1.37281300	−0.73949700	−0.02634600
C	−2.53523400	−1.33904100	0.43944600
C	−3.71184800	−0.62102400	0.45990000
C	−3.74508000	0.69628300	0.04854400
C	−2.57438600	1.29286300	−0.37570000
C	−1.35983900	0.60459900	−0.43189400
H	−4.66240500	1.25968500	0.07053500
Cl	−2.64978600	2.93979600	−0.86969400
H	2.26632100	2.13691000	1.54505100
H	−2.52077400	−2.36416300	0.77244000
S	0.10480000	1.40041800	−0.93063100
S	−0.01452500	−1.83767200	−0.23067200
Cl	5.00535600	1.69672000	0.92856000
Cl	−5.15835000	−1.37212900	1.01833900

TS19

C	1.36406400	0.71725100	0.40490600
C	2.53276200	1.35659800	0.02352900
C	3.63780800	0.64330100	−0.40995200
C	3.57132100	−0.73891300	−0.46085000
C	2.42398700	−1.41323300	−0.11725200
C	1.28649800	−0.68119800	0.25199300
Cl	2.66317000	3.06926400	0.12936000
H	4.53922500	1.16187300	−0.68854600
H	0.48776800	−0.64272100	−1.34920300
C	−1.35834800	0.69119100	0.41344700
C	−2.43923700	1.41192600	−0.06700200
C	−3.58125100	0.73462700	−0.44582800
C	−3.65158900	−0.64281100	−0.38303700
C	−2.55111900	−1.34991900	0.06309200
C	−1.39327900	−0.69949600	0.47567500
H	−4.54362900	−1.16421000	−0.68590100
Cl	−2.65654200	−3.06559700	0.12284200
H	2.37652500	−2.48814600	−0.17013800
H	−2.39195700	2.48605700	−0.13727800
S	−0.00177300	−1.58218100	1.07380000
S	0.00380300	1.61196800	1.04495600
Cl	−4.94164400	1.62155700	−1.01940400
Cl	4.96148000	−1.62554100	−0.95993600

TS20

C	−1.34910100	−0.56390900	−0.66847200

C	−1.78198600	−0.62701600	0.70009400
C	−3.07099800	−0.32640100	1.06136400
C	−4.01446100	−0.06974100	0.07622700
C	−3.68197700	−0.14476200	−1.27838800
C	−2.39736600	−0.42318700	−1.63769300
Cl	−0.64513600	−0.99273300	1.92106800
H	−3.35042400	−0.31063200	2.10144200
C	1.21132400	0.66464800	−0.43497700
C	2.31832000	1.38076700	0.00202900
C	3.53673900	0.76836000	0.23225100
C	3.64576400	−0.59361100	0.03964400
C	2.56340700	−1.34817900	−0.37365600
C	1.36388500	−0.70972000	−0.61951900
H	−4.44262500	0.00575300	−2.02655900
H	−2.11912200	−0.48196900	−2.67755800
H	4.38011800	1.34938600	0.56463700
H	2.65742500	−2.41505800	−0.49300700
Cl	2.18659300	3.08030100	0.24386500
S	−0.32810400	1.39648300	−0.75710500
S	−0.00964500	−1.61040600	−1.22510900
Cl	5.15499800	−1.37287300	0.34043600
Cl	−5.62484900	0.29893900	0.53304500

TS21

C	−1.22449000	0.38239600	−0.00934800
C	−1.33563300	−0.50003000	−1.13614300
C	−2.50135000	−1.13151200	−1.45400600
C	−3.65138900	−0.84668600	−0.71899400
C	−3.63416100	0.09246600	0.30567100
C	−2.46461200	0.73231800	0.62396200
H	−2.54548300	−1.82952300	−2.27348100
C	1.29732100	−0.58746900	0.66493400
C	2.38525100	−1.43950800	0.77258300
C	3.58777300	−1.07513900	0.19634800
C	3.72679800	0.10463900	−0.50698000
C	2.62690200	0.93433700	−0.62989700
C	1.41133400	0.60944800	−0.05026500
H	2.29916300	−2.37199100	1.30498100
H	−4.53895900	0.33081600	0.83929600
Cl	−2.47107000	1.88360900	1.88816500
H	−0.43917600	−0.69016200	−1.70636400
H	4.66528100	0.37395900	−0.96016600

Cl	2.79504000	2.40730200	−1.50657000
S	0.02033500	1.66569500	−0.12062400
S	−0.22328400	−0.94241700	1.42805000
Cl	−5.12329500	−1.63389400	−1.10569300
Cl	4.94425900	−2.12978700	0.34349300

TS22

C	1.36378800	0.58644500	−0.10944900
C	2.62614700	1.01549400	0.27501200
C	3.73836000	0.21381000	0.11543300
C	3.59959800	−1.05021800	−0.46780600
C	2.38370900	−1.51026800	−0.87525100
C	1.21865600	−0.73411300	−0.62841200
Cl	2.82993800	2.58116200	0.95939200
H	4.70645800	0.56642000	0.42847000
C	−1.34766700	−0.80564500	0.51731700
C	−2.51515100	−1.56081100	0.52012400
C	−3.70284600	−1.00188000	0.10039500
C	−3.75318800	0.29539200	−0.36856700
C	−2.58727700	1.03368200	−0.40054000
C	−1.37007200	0.51685200	0.04558500
H	−2.48425400	−2.58560400	0.85137400
H	2.28625400	−2.47101800	−1.35222900
H	0.36993700	−0.91489300	−1.27331800
H	−4.67814500	0.72817900	−0.70909600
Cl	−2.68824500	2.65351000	−0.98026800
S	−0.00667400	1.61964700	0.14451100
S	0.11266500	−1.56750800	1.09082100
Cl	5.01407100	−2.00557000	−0.70149300
Cl	−5.14520200	−1.94379200	0.13098400

TS23

C	1.35608700	0.48892500	0.36122100
C	2.52409100	1.08349800	−0.09123200
C	3.62958400	0.32814000	−0.44545600
C	3.56791200	−1.05121600	−0.34086600
C	2.42342800	−1.68528000	0.07944000
C	1.28485600	−0.91806000	0.36394400
Cl	2.65641900	2.79675500	−0.17548400
H	4.52860200	0.81485600	−0.78294000
H	0.49636200	−1.06327000	−1.23333000
C	−1.39426300	−0.89302100	0.57209300

C	−2.50167700	−1.64493200	0.22166300
C	−3.61592300	−1.00090900	−0.27780500
C	−3.62540900	0.36661800	−0.46544900
C	−2.49825500	1.09858200	−0.14201100
C	−1.36596400	0.48782500	0.39141200
H	−2.49296700	−2.71589800	0.33853700
H	2.37852900	−2.75936700	0.14854200
H	−4.49411900	0.86212400	−0.86424800
Cl	−2.54760400	2.80031700	−0.38877800
S	0.00582400	1.46258400	0.90419800
S	−0.00962900	−1.70614000	1.28179400
Cl	−5.01330600	−1.91865700	−0.69164000
Cl	4.95965000	−1.98444900	−0.74081200

TS24

C	−1.37433900	0.46404400	−0.51933300
C	−2.42122800	0.60963200	−1.48910200
C	−3.70342300	0.22750800	−1.23188900
C	−4.03459600	−0.25094200	0.03803000
C	−3.09381100	−0.29231800	1.05690400
C	−1.80733500	0.11387900	0.80485200
H	−4.46325200	0.30128400	−1.99220300
C	1.19089800	−0.76864500	−0.63088300
C	2.26500300	−1.61604600	−0.40425100
C	3.47815500	−1.07886900	−0.01860300
C	3.64260000	0.27861800	0.16943200
C	2.55564400	1.10902400	−0.03417800
C	1.32980300	0.60585600	−0.43417200
H	2.15951100	−2.68052500	−0.53135600
H	−3.37344400	−0.61771900	2.04480600
Cl	−0.67768300	0.11705100	2.08565500
H	−2.14314100	0.97811400	−2.46332300
H	4.58997200	0.68701500	0.47662600
Cl	2.75112300	2.80413600	0.20394800
S	−0.04593100	1.64393500	−0.73284800
S	−0.34029300	−1.37653100	−1.18185100
Cl	4.81773800	−2.13214400	0.24841900
Cl	−5.64143100	−0.75122300	0.35961400

TS25

C	−1.46141800	1.04157500	−0.23553700
C	−2.53240000	1.48171300	−0.99995300
C	−3.75883000	0.85967100	−0.94158600

C	−3.93126700	−0.24662400	−0.09567300
C	−2.91771800	−0.70759300	0.68404700
C	−1.61889600	−0.12160700	0.58545000
H	−4.58308300	1.20831800	−1.54097100
C	1.04286100	−0.71077600	−0.49802500
C	2.10941000	−1.60276500	−0.43482700
C	3.38746900	−1.14516500	−0.20372000
C	3.63418600	0.19645200	0.00939800
C	2.57013900	1.07236000	−0.01838200
C	1.26609800	0.65453300	−0.28053800
H	1.92767200	−2.65679800	−0.56501300
H	−3.05944600	−1.53212400	1.36180000
Cl	−0.67937600	−0.23018000	2.04785500
H	−2.38577400	2.31783600	−1.66544100
H	4.63010000	0.55715000	0.20118200
Cl	2.89795800	2.74410900	0.24960000
S	0.03930400	1.90830600	−0.35431500
S	−0.51982900	−1.39621800	−0.85643100
Cl	−5.47939000	−0.99776800	−0.01876700
Cl	4.69734400	−2.26430400	−0.15183800

TS26

C	1.36630700	0.71493700	0.49729800
C	2.50848700	1.35799300	0.03333400
C	3.57883500	0.64081400	−0.46847000
C	3.49832500	−0.73736000	−0.52578200
C	2.37583300	−1.40751700	−0.08031400
C	1.32403300	−0.67183400	0.43522900
H	4.45868700	1.15583400	−0.81489100
C	−1.36206600	−0.68598600	0.51370300
C	−2.47997000	−1.33435700	−0.00125900
C	−3.55256700	−0.62260000	−0.50448400
C	−3.49461800	0.75675500	−0.51492500
C	−2.38702600	1.43060700	−0.04142400
C	−1.33023300	0.70467400	0.48431000
H	2.32256400	−2.48328000	−0.12676500
S	0.01413200	1.61143700	1.16961100
Cl	4.82465600	−1.63068400	−1.16707800
Cl	2.63230800	3.07398200	0.09098400
Cl	−2.58030700	−3.05068500	−0.00032200
Cl	−4.82142700	1.64943600	−1.15228800
H	−4.41528400	−1.14174300	−0.88577700
H	−2.34254600	2.50637400	−0.08100300

S	−0.02723000	−1.59009000	1.22341800
H	−1.02476900	−1.97033000	2.54461200

TS27

C	1.75154500	−1.21020000	0.81195000
C	1.75803200	−1.96183000	−0.39815700
C	0.59028000	−2.33768000	−0.96127100
C	−0.67780300	−2.00547400	−0.37948900
C	−0.63304000	−0.98538600	0.67112700
C	0.59883300	−0.78264700	1.36925800
H	2.70343500	−2.25286500	−0.82732400
H	2.39416300	1.96019800	1.10937300
C	1.48596100	1.95899200	0.52310300
C	1.51650500	1.41555600	−0.82489300
C	0.26422700	2.18495300	0.98873000
C	0.42746800	0.78324800	−1.30215000
C	−0.90789900	1.83387800	0.44891900
C	−0.80002800	0.72147100	−0.51574000
H	0.58856200	−0.24423600	2.30163000
H	0.45084500	0.26825800	−2.24711300
S	−2.06033300	−2.71088300	−0.90758400
Cl	3.00047900	1.39778400	−1.69305600
Cl	3.25961700	−0.89156200	1.57335200
Cl	−2.04839200	−0.80876700	1.65435300
Cl	−2.20926300	0.40736600	−1.46021100
H	0.57669800	−2.96592100	−1.83722200
S	−2.31256700	2.79479700	0.77810100
H	−3.03328500	2.42070200	−0.28138900

TS28

C	1.54070900	−0.93579500	−1.21220800
C	2.31232900	−0.14375500	−0.32181900
C	1.93798800	1.12885400	−0.04408400
C	0.76401900	1.70691300	−0.64375200
C	−0.13092700	0.75963300	−1.28185500
C	0.36568100	−0.49096400	−1.72203000
H	3.21060500	−0.55554300	0.10814500
H	−0.48133400	−3.29913500	−0.42800000
C	−0.97729200	−2.44317800	0.00975400
C	−0.34924300	−1.76127700	1.12320800
C	−2.02776500	−1.81375900	−0.51478600
C	−0.52444000	−0.43072400	1.25680800
C	−2.42746600	−0.55802900	−0.27014100

C	−1.39669100	0.28675600	0.34440900
H	−0.20800500	−1.09440000	−2.40448500
H	0.01762300	0.13553400	1.99570600
S	0.40382000	3.30871100	−0.61466200
Cl	0.75762600	−2.61230200	2.12643900
Cl	2.15368500	−2.48411600	−1.63399400
Cl	−1.89669200	1.83148700	0.91617200
S	−4.08631800	−0.12198400	−0.55590600
H	−4.14461500	0.91832600	0.27962000
H	−0.94021000	1.20920500	−1.83882300
Cl	2.92329200	2.08083300	0.98408200

TS29

C	2.51664200	−1.42262900	−0.80123600
C	3.34706400	−0.34781400	−0.35007700
C	2.82916200	0.86234400	−0.05260500
C	1.41682700	1.15024300	−0.18057000
C	0.61628500	0.10032600	−0.90130800
C	1.21249200	−1.24547400	−1.01966600
H	4.40131800	−0.52743700	−0.21534800
H	0.64451000	0.51018000	−1.98576600
Cl	3.87412500	2.07732800	0.55345600
H	−4.57964300	−0.26725000	0.36134300
C	−3.53700400	−0.17696200	0.10793300
C	−3.14538300	0.20596000	−1.15590900
C	−2.56593900	−0.43356500	1.05806600
C	−1.80704100	0.32104200	−1.47697900
C	−1.20901700	−0.33652800	0.76613200
Cl	−3.09915200	−0.89752600	2.62966300
C	−0.84107700	0.04730400	−0.52794200
H	−1.52068500	0.62532100	−2.47068800
H	0.58182700	−2.05395400	−1.35266000
S	0.05252300	−0.60148900	1.96349200
S	0.72655600	2.53677800	0.32191000
Cl	3.26694500	−2.95823500	−1.01554700
Cl	−4.34131600	0.54152200	−2.34960500
H	0.64205200	1.06001300	−3.17735000
H	−0.54294000	−1.62947700	2.57356200

TS30

C	−2.38399800	−1.46444000	−1.07228400
C	−3.27055100	−0.40493600	−0.76769600
C	−2.91730400	0.59294300	0.09076700

C	−1.65518400	0.58810200	0.72569400
C	−0.69835400	−0.45057800	0.35540700
C	−1.13873600	−1.48461400	−0.55701300
H	−4.25723000	−0.40319600	−1.20346700
H	−0.57126700	−0.80694600	1.50937800
Cl	−4.04726600	1.83278700	0.45327600
H	4.20677000	1.18865300	−1.09176100
C	3.22917900	0.86565500	−0.77695800
C	2.11352200	1.63382100	−1.02664200
C	3.07503100	−0.33162400	−0.10319900
C	0.86140700	1.21269300	−0.63073300
C	1.82862200	−0.78107400	0.33262800
Cl	4.49931500	−1.25355200	0.18881300
C	0.71294500	0.01009500	0.04026200
H	0.00053200	1.82278100	−0.85035100
H	−0.43469500	−2.25862900	−0.82023600
S	1.69454900	−2.32209600	1.18614600
S	−1.27667000	1.56528200	2.02841800
Cl	−2.93804200	−2.69683100	−2.14158300
Cl	2.28282800	3.13074200	−1.86315600
H	−0.66459800	−0.45516300	3.61857500
H	1.05208700	−1.78980400	2.24093800
O	−0.13791800	−0.29105000	2.83344300

TS31

C	−2.52031900	1.51748800	0.99648100
C	−3.40488600	0.45526100	0.74251200
C	−2.96796500	−0.66893100	0.09891700
C	−1.63448700	−0.80758900	−0.33731200
C	−0.71901900	0.29574500	−0.08583500
C	−1.21842500	1.43692300	0.63486400
H	−4.43585300	0.53228000	1.04700900
H	−0.57820000	0.50097000	−1.36810300
Cl	−4.09423400	−1.92955400	−0.20199600
H	4.40363600	−0.87842400	1.00014400
C	3.37656100	−0.63811700	0.78380800
C	2.35393700	−1.47941100	1.17166000
C	3.06172400	0.53158300	0.12468400
C	1.04223800	−1.17149400	0.88780900
C	1.74435500	0.87377500	−0.18846200
Cl	4.37272600	1.56362800	−0.31261600
C	0.73115600	−0.01163300	0.19660100

H	0.25687100	−1.84446600	1.18838400
H	−0.53733100	2.22579900	0.91392700
S	1.32222200	2.39365600	−0.97219400
S	−1.14626400	−2.02706200	−1.39308100
Cl	−3.12450000	2.90237600	1.82553000
Cl	2.72821900	−2.92990600	2.02173000
S	−0.01874900	−0.25080400	−2.89984300
H	−1.12512200	−0.19847600	−3.64749900
H	2.43386100	2.49683600	−1.70133700

TS32

C	3.12131100	1.18601300	0.40240300
C	3.52423000	0.03159500	−0.24286100
C	2.57414100	−0.87787800	−0.67343300
C	1.22103200	−0.65101000	−0.46463100
C	0.84770100	0.53287000	0.19024000
C	1.78200600	1.45222500	0.62314300
H	4.57021800	−0.16380800	−0.41080300
H	0.44816800	−1.19088000	1.98772700
Cl	3.08591400	−2.29947100	−1.49395300
H	−4.39115800	0.40485200	−0.49045100
C	−3.33861000	0.46901700	−0.27077400
C	−2.64713600	1.65354800	−0.48840500
C	−2.68200800	−0.61159400	0.25781700
C	−1.27644000	1.73843900	−0.25345500
C	−1.28726000	−0.57472100	0.57015900
Cl	−3.56470800	−2.04522100	0.57483700
C	−0.60558500	0.65996000	0.25333800
H	1.47815500	2.35129800	1.13473300
H	−0.73503000	2.62707000	−0.53595300
S	−0.06125700	−1.70818500	−0.97983800
S	−0.85906700	−1.45612600	2.06494300
Cl	−3.48394200	3.01111700	−1.11491800
Cl	4.31300100	2.30708500	0.94767100

TS33

C	3.11664600	1.37162800	−0.00990000
C	3.58935500	0.07456400	−0.16287300
C	2.68897300	−0.96677600	−0.25148200
C	1.32900800	−0.71711900	−0.18741800
C	0.87002100	0.59474300	−0.03442200
C	1.76960000	1.64940900	0.05292900
H	4.64775800	−0.11879700	−0.21081900

H	−2.03591700	0.20063100	2.33177600
Cl	3.25251800	−2.57795800	−0.45218800
H	−4.35174100	0.17353200	−0.65611000
C	−3.30296100	0.28400000	−0.43798200
C	−2.74311100	1.56228100	−0.30206200
C	−2.50519900	−0.81398400	−0.31176000
C	−1.38209200	1.75378800	−0.13740100
C	−1.13729600	−0.67127400	0.10194900
Cl	−3.13630000	−2.38777900	−0.51385000
C	−0.56578500	0.64955200	−0.02935900
H	1.43030600	2.66467500	0.17723300
H	−0.97472000	2.75143700	−0.15052100
S	−1.26965200	−0.89306900	2.23770100
S	0.07114200	−1.91051600	−0.30896200
Cl	4.25632200	2.66084000	0.10574200
Cl	−3.77856900	2.92613300	−0.43927400

TS34

C	2.24264900	1.49868000	−0.68952900
C	3.11805800	0.44137700	−0.62178900
C	2.64538500	−0.78150900	−0.18923900
C	1.31170300	−0.99110700	0.17324200
C	0.45235400	0.12354000	0.15251400
C	0.92882500	1.34486600	−0.30366800
H	4.15173500	0.55374200	−0.90119200
Cl	3.80451600	−2.06044500	−0.13459900
H	−1.20849200	−1.65740600	−0.91785300
C	−1.70101500	−0.75626200	−0.58987800
C	−2.87273900	−0.37715500	−1.10600900
C	−1.02873200	0.03919500	0.45744400
C	−3.54082100	0.81530000	−0.67467700
C	−1.70022000	1.34692700	0.83020300
Cl	−1.31642900	−0.92899100	2.08373700
C	−2.97680700	1.62117900	0.24491100
H	−3.47438300	2.51989800	0.57099500
H	−1.20562900	−2.08234800	3.72837900
H	0.26979600	2.19053400	−0.37622800
H	−4.50099800	1.05447500	−1.10321200
S	−1.05109100	2.36832100	1.92963600
S	0.74560800	−2.59937000	0.62504000
Cl	2.79232900	3.02933000	−1.25543200

TS35

C	2.54869200	1.52482300	−0.38726500
C	3.29623200	0.37628300	−0.50279200
C	2.65774100	−0.84392500	−0.40234700
C	1.28088700	−0.95795500	−0.19257400
C	0.55088500	0.23160700	−0.01786400
C	1.19437900	1.45503600	−0.14444900
H	4.35830300	0.41746900	−0.67496700
Cl	3.66006400	−2.24010500	−0.56443000
H	−1.18046400	−0.98540800	−1.60724700
C	−1.65310400	−0.22679100	−1.00600500
C	−2.88263200	0.22878200	−1.31683500
C	−0.93953700	0.27697600	0.14782600
C	−3.52353500	1.23756900	−0.54796500
C	−1.55172100	1.42230500	0.87123000
Cl	−1.44291900	−1.06149600	1.66998600
C	−2.87467200	1.80157000	0.49665300
H	−3.34078700	2.57874800	1.07972400
H	−0.56101700	−3.16806800	2.53835100
H	0.63370700	2.36966800	−0.08025200
H	−4.51912300	1.55113400	−0.81698300
S	0.49133700	−2.53429200	−0.16196600
S	−0.81017500	2.19033900	2.13841900
Cl	−3.71412100	−0.38525300	−2.68872500
Cl	3.31075800	3.05986800	−0.54655500
H	1.42996400	−3.14402700	−0.88879600
O	−1.39689700	−2.82689700	2.88081700

TS36

C	2.79231300	−1.41749700	−0.08107500
C	3.42933400	−0.27006300	0.32955000
C	2.66844100	0.84952900	0.60027100
C	1.27592000	0.86299100	0.47960900
C	0.65790300	−0.30317500	−0.00589900
C	1.42557800	−1.43416500	−0.25162100
H	4.49923500	−0.23817800	0.44639900
Cl	3.53812400	2.24880800	1.11692600
H	−1.07013900	0.17861200	1.93759800
C	−1.49576800	−0.43729100	1.16416900
C	−2.61117900	−1.15033900	1.38882100
C	−0.83161000	−0.46742100	−0.13126500
C	−3.18665800	−1.98361200	0.38358900
C	−1.34963900	−1.45743800	−1.12846200
Cl	−1.59305800	1.13516500	−1.12949600

C	−2.57448500	−2.12166900	−0.81165400
H	−2.98982800	−2.76147000	−1.57280100
H	−0.73737900	3.80887700	−1.80517500
H	0.95561100	−2.35128300	−0.55595800
H	−4.10390200	−2.50675900	0.60082300
S	0.33780600	2.28598000	0.92632000
S	−0.63038200	−1.71443100	−2.58835100
Cl	−3.38325700	−1.10283600	2.92336900
Cl	3.70826600	−2.84251600	−0.38737300
H	1.24785400	2.74954300	1.78568600
S	−2.05702500	3.59376400	−1.79740700

TS37

C	2.80757800	−1.39143200	−0.02945100
C	3.43094600	−0.22559600	0.34971800
C	2.66115200	0.89921600	0.57024900
C	1.27045300	0.90036700	0.43097300
C	0.66671600	−0.28949700	−0.01341100
C	1.44261400	−1.42460500	−0.21220500
H	4.49910100	−0.18273800	0.47897300
Cl	3.51246800	2.32096700	1.04842200
H	−1.07485200	0.30591500	1.88980000
C	−1.48886100	−0.36768900	1.15795600
C	−2.59131400	−1.08795600	1.42354400
C	−0.81969700	−0.46645200	−0.13128700
C	−3.14944800	−1.98949100	0.46853700
C	−1.33209500	−1.50874000	−1.07689600
Cl	−1.60389300	1.04269200	−1.23089300
C	−2.53932200	−2.18109100	−0.72132600
H	−2.94558300	−2.86807600	−1.44526000
H	0.98166500	−2.35676500	−0.48372000
H	−4.05494900	−2.51869300	0.71809100
S	−0.61231800	−1.81801800	−2.53017900
S	0.31713200	2.33664000	0.78040500
Cl	3.73665900	−2.81885500	−0.27700200
Cl	−3.36408000	−0.96764400	2.95175500
H	1.19686600	2.84025400	1.64883200
Cl	−2.04771800	3.43930800	−1.79279600

TS38

C	−3.55060200	0.35678200	−0.08858600
C	−3.70854500	−0.69823000	0.79470200
C	−2.60024300	−1.35605100	1.29732500

C	−1.32631000	−0.95250400	0.92319400
C	−1.18723000	0.11449400	0.02447800
C	−2.29237000	0.77287700	−0.48178500
H	−4.70031400	−1.00770400	1.08252700
H	−0.63354500	−1.89880100	−1.39634500
H	0.07584100	2.53001400	0.14160100
C	0.73481100	1.68966100	−0.00723600
C	2.11336400	1.85850500	0.09554700
C	0.21576200	0.44010800	−0.20898800
C	2.97376800	0.76909700	0.05306700
C	1.06844800	−0.72104000	−0.33870200
C	2.46818600	−0.48464600	−0.17179500
H	−2.17885000	1.58498600	−1.18242600
H	4.03534500	0.90914700	0.16995200
S	0.68903000	−1.95212000	−1.57904000
S	0.15194800	−1.66321100	1.51979700
Cl	2.75808100	3.42808200	0.33982700
Cl	−4.94511800	1.15413200	−0.72146500
H	−2.73025500	−2.17784000	1.98340300
Cl	3.55303000	−1.80744000	−0.27707700

TS39

C	3.60158100	0.35190000	−0.10289900
C	3.77585500	−1.00835900	−0.33450400
C	2.67766000	−1.83533700	−0.45163600
C	1.41056200	−1.28869000	−0.34070500
C	1.23685600	0.07788200	−0.10291700
C	2.34757100	0.90658800	0.01425300
H	4.77270100	−1.40978000	−0.41618900
H	−1.64045400	0.16203900	2.33345200
H	−0.11013700	2.58334600	−0.02509800
C	−0.71992100	1.69530100	−0.05877600
C	−2.09398500	1.80648800	−0.18727900
C	−0.15268000	0.43933400	−0.04580200
C	−2.91630000	0.68798600	−0.38312700
C	−0.99325900	−0.73564700	0.02730700
C	−2.36727300	−0.55898300	−0.35112700
H	2.23741100	1.96232200	0.20341100
H	−3.96850700	0.81692500	−0.57261000
S	−1.13235900	−1.06258400	2.14757300
S	−0.08504700	−2.16862400	−0.49938100
Cl	−2.81708500	3.36463800	−0.20320800
Cl	4.99320100	1.36231800	0.04782400

Cl	−3.32115100	−1.94787300	−0.63075000
H	2.81309200	−2.89073500	−0.62648800

TS40

C	1.33750400	−0.81486600	−1.34960700
C	2.16645100	0.02515600	−0.55148200
C	1.74772500	1.26520700	−0.19979500
C	0.45694000	1.75390800	−0.60727400
C	−0.46725300	0.73126300	−1.09393300
C	0.07536100	−0.46323600	−1.66989700
H	3.14581100	−0.32206600	−0.26564100
H	−0.52400400	−3.66888700	0.42607500
C	−0.81243900	−2.63159600	0.49583500
C	−0.02592700	−1.71755800	1.27322900
C	−1.90331000	−2.12665700	−0.09220800
C	−0.35037200	−0.41116000	1.36437100
C	−2.36217100	−0.80061200	−0.10192500
C	−1.40216200	0.13941400	0.54262800
H	−0.54680100	−1.09991100	−2.27660300
H	0.21160100	0.25931100	1.99304900
S	−0.00219900	3.32477200	−0.53565000
Cl	1.33096500	−2.34499900	2.12104300
Cl	2.00822300	−2.30381300	−1.88796400
Cl	−2.11701900	1.57255900	1.18691100
S	−3.75618600	−0.35770600	−0.84734200
H	−1.36723300	1.13351700	−1.54315700
Cl	2.80446100	2.28386600	0.68203900

TS41

C	−1.59153400	1.09147900	1.05774600
C	−1.66372800	2.00458800	−0.04288200
C	−0.53859200	2.38853300	−0.67520000
C	0.76008600	1.91253400	−0.28445600
C	0.76273700	0.74859400	0.64031200
C	−0.42997500	0.53928000	1.43852900
H	−2.62577100	2.39913600	−0.32825000
H	−2.62641000	−2.45148100	0.41054400
C	−1.67601400	−2.05525900	0.08905300
C	−1.61068000	−1.14168400	−1.02303000
C	−0.50917200	−2.36995100	0.65175000
C	−0.45315500	−0.57521100	−1.40690400
C	0.78169400	−1.93375200	0.29627400
C	0.75899600	−0.75923200	−0.62683700

H	−0.36834500	−0.11800900	2.28953200
H	−0.41154700	0.08049100	−2.25980900
S	2.11569900	2.61541100	−0.85912000
S	2.12761800	−2.66125100	0.86641900
Cl	−3.07359300	−0.79462100	−1.85809900
Cl	−3.04989000	0.73045800	1.89483200
Cl	2.22165500	0.54337700	1.54724600
Cl	2.20566300	−0.56166600	−1.55183800
H	−0.57017500	3.12547000	−1.46133300

TS42

C	−2.76578100	1.33912800	−0.73575000
C	−3.45211200	0.41232600	0.07262400
C	−2.80078500	−0.65892600	0.61039900
C	−1.42604300	−0.89478600	0.37373300
C	−0.71327300	0.08157600	−0.44175800
C	−1.43956800	1.20663400	−0.96035100
H	−4.50700400	0.54083400	0.25293500
H	−0.40728400	−0.82785500	−1.33374400
Cl	−3.68911000	−1.76272100	1.58002300
H	4.49900800	0.78315700	0.46351300
C	3.44385900	0.65520100	0.28967300
C	2.54335400	1.62808300	0.68415800
C	2.97576700	−0.48673200	−0.33112800
C	1.18374900	1.48469200	0.47247400
C	1.61667800	−0.65818500	−0.56530500
Cl	4.09997100	−1.68804000	−0.82217500
C	0.72893000	0.33811000	−0.14872400
H	−0.90320200	1.94176100	−1.53922200
H	0.49805100	2.24871300	0.80099300
S	−0.66962900	−2.33089800	0.79766000
S	0.87899600	−2.02312700	−1.32777300
Cl	3.13221200	3.04759300	1.46427400
Cl	−3.65472700	2.65581800	−1.40347200

TS43

C	2.48106800	−1.23361500	1.10224500
C	3.38261600	−0.27989400	0.58780600
C	2.93444100	0.78410400	−0.13225400
C	1.53935700	1.01782500	−0.42689900
C	0.67168400	−0.06525800	0.00374900
C	1.15687600	−1.11083000	0.85417800
H	4.43788800	−0.38719800	0.78084500

H	1.08207100	−0.65863700	−1.24276500
Cl	4.09900300	1.91674500	−0.68382900
H	−4.61474000	0.69243400	0.54847200
C	−3.56143100	0.52448400	0.40224500
C	−2.63182100	1.49271600	0.76462000
C	−3.11094100	−0.64962200	−0.13553000
C	−1.27920800	1.29929200	0.58270500
C	−1.72325200	−0.89692600	−0.36019100
C	−0.80341800	0.12495900	0.03026300
H	−0.58344900	2.06706800	0.87400700
H	0.45755100	−1.82064500	1.26623200
O	1.32343400	−1.28132600	−2.23608800
H	2.20459600	−1.66135300	−2.19662600
H	0.64248600	−1.97663500	−2.11832300
S	−1.26062300	−2.36000000	−1.08455900
S	1.02451600	2.31580300	−1.33242000
Cl	−4.28859800	−1.82342900	−0.55392100
Cl	−3.18938100	2.94741800	1.47426800
Cl	3.09827100	−2.53136300	2.05824100

TS44

C	−1.76526400	−1.25319900	−0.77364300
C	−1.77604400	−1.98792200	0.45775300
C	−0.57975900	−2.28768600	0.96653500
C	0.69157700	−2.00870400	0.43002700
C	0.63242700	−1.00698800	−0.64604100
C	−0.61069300	−0.82937600	−1.33577800
H	−2.71180800	−2.27950700	0.90841900
H	−2.40778800	1.91961400	−1.11804300
C	−1.49586000	1.93184300	−0.53766300
C	−1.51593400	1.41154600	0.81970800
C	−0.27801500	2.15312400	−1.01470600
C	−0.42079400	0.79358300	1.30242900
C	0.90009900	1.81562100	−0.47900000
C	0.80173400	0.72385000	0.50981100
H	−0.61037100	−0.30413000	−2.27555400
H	−0.43618100	0.29459400	2.25634000
S	2.05771900	−2.72148400	0.98861900
Cl	−2.99597500	1.39783600	1.69303700
Cl	−3.27289800	−0.94582600	−1.53991900
Cl	2.04483600	−0.85563900	−1.63544000
Cl	2.21837200	0.42846200	1.44829400
S	2.30039000	2.77132700	−0.83915900

H	3.03185100	2.41563500	0.21934700

TS45

C	−1.65902800	−1.35363800	−0.67473500
C	−1.57124600	−1.84152800	0.68378000
C	−0.34402700	−2.15691200	1.09832900
C	0.80008700	−1.94159100	0.42369600
C	0.71034200	−0.90086500	−0.59433100
C	−0.54891100	−0.87291000	−1.28627400
H	−2.44527500	−1.76358700	1.31578700
H	−2.44587700	1.76305100	−1.31576800
C	−1.57183000	1.84118900	−0.68381600
C	−1.65942700	1.35325700	0.67471000
C	−0.34464400	2.15650900	−1.09846200
C	−0.54920300	0.87276900	1.28625300
C	0.79949800	1.94165500	−0.42373000
C	0.71003700	0.90086000	0.59427900
H	−0.59956000	−0.40216000	−2.25307400
H	−0.59978600	0.40198800	2.25304300
Cl	−3.20200100	1.19811100	1.41303600
Cl	−3.20166100	−1.19876800	−1.41300200
Cl	2.10128100	−0.61357100	−1.59681500
Cl	2.10101900	0.61414500	1.59687700
S	2.16375700	−2.97152500	0.71232200
H	2.82999300	−2.69452900	−0.41129700
S	2.16279000	2.97205400	−0.71231700
H	2.82902700	2.69543100	0.41139800

TS46

C	−2.74296000	1.36935200	−0.84981300
C	−3.50713400	0.44374600	−0.17119200
C	−2.87280400	−0.54871500	0.55261700
C	−1.48184700	−0.62624900	0.63689700
C	−0.73180400	0.32838200	−0.06507900
C	−1.36346300	1.31408100	−0.80667200
H	−4.58271900	0.48187000	−0.20851700
H	−0.39671400	−1.36634700	−1.73273500
Cl	−3.86925500	−1.69421700	1.36266500
H	4.59491100	0.47236700	0.27380700
C	3.52118200	0.44382600	0.19626000
C	2.73542000	1.35432200	0.87065600
C	2.90856900	−0.50875400	−0.59271700
C	1.36005500	1.30524500	0.77003100

C	1.52414500	−0.58399300	−0.72421000
Cl	3.90583000	−1.63072900	−1.43741500
C	0.74996500	0.33441100	−0.00777000
H	−1.43236200	−1.79440400	2.68764600
H	−2.03682900	−1.56731000	3.78969300
H	0.75323800	2.01160800	1.31286900
H	−0.77414100	2.03853300	−1.34497500
S	0.85730800	−1.82131200	−1.77542300
S	−0.64815900	−1.88633300	1.53256700
Cl	−3.52111500	2.60522900	−1.76134300
Cl	3.48055700	2.56042800	1.85102600

TS47

C	−2.60755700	1.75435900	−0.55477000
C	−3.41287100	0.66926300	−0.27769200
C	−2.82798500	−0.53253500	0.07447500
C	−1.44219800	−0.66704100	0.17902900
C	−0.64799500	0.45083600	−0.11233300
C	−1.23191900	1.65160400	−0.48084600
H	−4.48503700	0.74831800	−0.34030600
H	−0.26183600	−0.53988100	−2.25868500
Cl	−3.87190700	−1.85998100	0.38534000
H	4.65667500	0.29626000	0.46989600
C	3.58760500	0.33067900	0.34445100
C	2.78256600	0.95271300	1.27547400
C	3.00150200	−0.24604000	−0.76422100
C	1.41332500	0.98705300	1.10812500
C	1.62403100	−0.22550000	−0.96982300
Cl	4.02298300	−1.00938800	−1.92178600
C	0.82914600	0.39071300	0.00195000
H	−1.59147500	−2.59409500	1.54293100
H	0.78986500	1.46465300	1.84657400
H	−0.61042800	2.50318000	−0.70515600
S	0.99009600	−0.97204900	−2.42590500
S	−0.65779400	−2.17320600	0.60712200
H	−0.97232500	−2.13684400	3.43956800
Cl	−3.32934900	3.25160000	−1.00004700
Cl	3.49576200	1.68834200	2.66162100
O	−1.83068500	−2.21423400	3.00445300

TS48

C	−2.37987600	2.13828400	0.27156400
C	−3.25654200	1.13579000	−0.08852400

C	−2.75391300	−0.11314100	−0.39931000
C	−1.38456100	−0.39137200	−0.33741500
C	−0.51769800	0.65029600	0.03332300
C	−1.01787600	1.90651300	0.32999600
H	−4.31650400	1.31971100	−0.13504600
H	−0.03875600	0.81075600	−2.32932600
Cl	−3.87751900	−1.32952500	−0.85018800
H	4.73079200	−0.13442700	0.61144500
C	3.67352500	0.03014800	0.48954900
C	2.85251900	0.21042400	1.58208300
C	3.11950600	0.07140000	−0.77465600
C	1.49743700	0.41478600	1.41644700
C	1.75958800	0.28342800	−0.98034900
Cl	4.16533800	−0.14747800	−2.12536600
C	0.94436300	0.43458800	0.14584100
H	−1.56656700	−2.73965400	0.18814200
H	0.86017500	0.53936800	2.27702300
H	−0.34444900	2.70151000	0.60623700
S	1.16847200	0.32942900	−2.63287700
S	−0.73491600	−1.95903000	−0.74834700
H	−0.92173900	−3.49667500	2.14569000
S	−2.15557200	−3.39471100	1.64058500
Cl	−2.99333700	3.69875200	0.65150500
Cl	3.52495700	0.17599000	3.16844600

TS49

C	2.46222700	−1.99547200	0.58211400
C	3.31084300	−1.02966200	0.07846100
C	2.77690900	0.14863500	−0.40404700
C	1.39654500	0.38556500	−0.37570400
C	0.55496800	−0.61944600	0.13457600
C	1.09106500	−1.80179800	0.60689700
H	4.37546600	−1.18957200	0.05597500
H	0.09114500	−1.24201700	−2.14597300
Cl	3.86060300	1.31235600	−1.04037700
H	−4.70792800	0.14745000	0.55869100
C	−3.64710000	−0.01545600	0.46955400
C	−2.82627400	0.03651600	1.57687300
C	−3.09009900	−0.28603000	−0.76404900
C	−1.46639800	−0.16447100	1.45274400
C	−1.72471800	−0.50554100	−0.92585100
Cl	−4.13243500	−0.35362100	−2.13260800

C	−0.91231800	−0.42159700	0.20914100
H	1.64874600	2.75192800	−0.42934600
H	−0.82868500	−0.09824000	2.31926000
H	0.44219900	−2.56877500	0.99706800
S	−1.12426900	−0.85512500	−2.53889300
S	0.68146200	1.85365300	−0.95894200
Cl	1.74622500	3.54424400	1.14640500
Cl	−3.50612900	0.36104000	3.12460900
Cl	3.12056300	−3.46194100	1.18229200

Table S5. Cartesian coordinates for the reactants, intermediates and products involved in the formation of PCTA/DTs from R1, R2 and DR.

IM1

C	−1.12973500	0.18337700	−1.00423400
C	−1.72079100	1.23998800	−0.31298000
C	−3.02883700	1.15299400	0.13090200
C	−3.75220900	0.00420000	−0.11954600
C	−3.20117300	−1.05167600	−0.82149300
C	−1.89887100	−0.94525800	−1.26447800
Cl	−0.87760900	2.70777000	−0.01314800
H	−3.47372700	1.97665700	0.66316800
H	−1.45622100	−1.75132200	−1.82648900
C	1.36987300	−0.78779200	−0.33966800
C	2.86543300	−0.57681000	−0.55023300
C	3.51732700	0.37844000	0.11422900
C	2.72977600	1.30572800	0.94143400
C	1.63557100	0.74286500	1.41019400
C	1.04710500	−0.40032200	1.09226800
Cl	1.04529100	−2.53143400	−0.64248800
H	3.35096300	−1.19923500	−1.28286200
H	2.95002700	2.36222000	0.91598000
H	−3.78520600	−1.93399900	−1.02405000
S	0.53430100	0.25690000	−1.58664400
Cl	−5.37763800	−0.10004600	0.44064700
Cl	5.19210000	0.67476800	−0.14014200
S	0.20414800	−1.33072100	2.28968100
H	0.30171400	−2.51558400	1.68452600

IM2

C	1.58676500	−0.97818800	−0.44046900
C	2.08337100	−0.55640900	0.79281000
C	3.36927600	−0.06151000	0.90880600
C	4.17542700	−0.00570400	−0.21251400
C	3.71539700	−0.42556600	−1.44609600
C	2.42271600	−0.90101700	−1.54719700
Cl	1.11352800	−0.62790700	2.20769500
H	3.73746900	0.26630300	1.86637000
H	2.04232300	−1.22329200	−2.50354400
C	−1.10729300	−0.37820900	−0.37991600
C	−0.69316500	0.94137500	−0.37896400
C	−1.62478600	1.94535900	−0.21080200
C	−2.96296600	1.65858000	−0.04943200

C	−3.36056100	0.33511800	−0.04196300
C	−2.45654400	−0.71026300	−0.20260700
H	−3.68810600	2.44390400	0.07754500
Cl	−5.04357000	0.02278500	0.18154400
H	4.35505100	−0.37386000	−2.31136600
H	0.34534600	1.19227600	−0.51835400
S	0.00610900	−1.72457200	−0.64233100
Cl	−1.10658500	3.59017400	−0.21453400
Cl	5.78052500	0.59856000	−0.05933400
S	−2.89033700	−2.41143800	−0.16128900
H	−4.17652400	−2.23198000	−0.46395700

IM3

C	0.99523000	−0.99038900	0.62656200
C	1.63629600	0.17283900	1.07260100
C	2.94648100	0.35581500	0.72165300
C	3.54623200	−0.61550500	−0.08574700
C	2.87412900	−1.73223300	−0.53096700
C	1.53904000	−1.98348800	−0.17018700
Cl	0.85529000	1.36980400	2.06616100
H	3.49794800	1.21804800	1.05488100
C	−1.68656000	−0.56603400	−0.51215300
C	−0.87032700	0.51733200	−1.08816600
C	−1.15523100	1.79045800	−0.81802900
C	−2.28668600	2.15595500	−0.00037600
C	−3.13578400	1.22571800	0.46614200
C	−2.97105300	−0.17630400	0.18654300
H	−2.44352200	3.20089100	0.21509800
H	3.41055200	−2.42745500	−1.16182100
H	−0.01932000	0.22700100	−1.68259000
S	−4.03438800	−1.29301100	0.69676200
Cl	−0.18848000	3.06507500	−1.43209300
Cl	5.20564900	−0.36501500	−0.51915500
Cl	−1.90043100	−1.84611900	−1.68873300
H	−3.99887900	1.50480700	1.04831500
S	−0.68623900	−1.33865800	0.94591400
H	−1.03185300	−0.34521200	1.79098700

IM4

C	1.68246500	−0.30144800	−0.76911800
C	2.40878000	−1.12937100	0.09591400
C	3.67736700	−0.74575000	0.44219600
C	4.15070000	0.46480400	−0.06664700

C	3.38821900	1.27717800	−0.87784400
C	2.09202100	0.91506100	−1.28165200
Cl	1.75691700	−2.60026800	0.75771900
H	4.28575900	−1.35288500	1.09018700
C	−0.89814400	1.27815400	0.32791200
C	−2.01986600	1.99565900	0.30199700
C	−3.30957500	1.37387400	0.16781900
C	−3.45282900	0.03576000	0.08292600
C	−2.31993800	−0.85622400	0.12898800
H	−4.18215900	2.00628900	0.13401900
Cl	−5.02327900	−0.62688000	−0.06473600
H	3.82034800	2.21291000	−1.20481500
H	0.07780000	1.73434100	0.39100500
S	−2.43512200	−2.48028000	0.08838500
Cl	−1.99889300	3.70737400	0.42729500
Cl	5.75742500	0.93795400	0.37912300
S	0.03343800	−0.69033900	−1.25635800
H	−0.10342400	−2.00266200	−0.96409900
C	−0.96915900	−0.19449900	0.23087500
H	−0.41852700	−0.65633000	1.05125900

IM5

C	1.42315000	−0.79211500	0.07827100
C	2.69610300	−0.93862100	−0.46037200
C	3.76709700	−0.20532600	0.01700400
C	3.56507800	0.69001400	1.04673600
C	2.31231000	0.84796400	1.61071800
C	1.25782200	0.09966900	1.13073300
Cl	2.98022900	−2.04878800	−1.74590400
H	4.74340500	−0.33213500	−0.41976400
H	0.28372600	0.21484100	1.58023600
C	−1.30781000	−0.79019900	−0.20095900
C	−2.28264900	−1.37066400	0.58212100
C	−3.47021100	−0.69262000	0.81753700
C	−3.68583600	0.57378000	0.30865700
C	−2.70570200	1.15982400	−0.46454800
C	−1.49140300	0.50035300	−0.77842200
H	−4.60365200	1.09844300	0.51270500
Cl	−3.02850800	2.73055300	−1.06515900
H	2.16638100	1.54190200	2.42202900
H	−2.12000200	−2.34311500	1.01645800
S	−0.33865900	1.19555500	−1.80742500
S	0.10500100	−1.78466100	−0.54231300

Cl	4.89449000	1.61460800	1.63779300
Cl	−4.68144700	−1.42852500	1.78264100

IM6

C	1.36652200	−0.83720300	0.06481500
C	2.62136600	−1.31581500	−0.20461300
C	3.76440800	−0.54463300	−0.01336700
C	3.62128000	0.76606100	0.48590700
C	2.41772600	1.30522000	0.77088600
C	1.17029400	0.55081400	0.53233600
Cl	2.81314900	−2.92283500	−0.79865100
H	4.73734000	−0.94598200	−0.23586600
H	0.54122900	0.56537000	1.42566700
C	−1.38564400	−0.72613300	−0.00544300
C	−2.57028100	−1.34834400	0.37040300
C	−3.74541300	−0.62951200	0.37562800
C	−3.76410800	0.70805600	0.03101700
C	−2.57527700	1.32420000	−0.30387400
C	−1.36368200	0.63503900	−0.32728700
H	−4.68331500	1.26853600	0.03629100
Cl	−2.62188400	2.99454900	−0.71741100
H	2.33517800	2.30364300	1.16749900
H	−2.57309700	−2.39189700	0.64124600
S	0.13197400	1.45320300	−0.71860200
S	−0.02836300	−1.82445600	−0.20606000
Cl	−5.21509700	−1.40677200	0.82425100
Cl	5.06158200	1.67705100	0.75659200

IM7

C	−1.27168100	−0.06147300	−0.55964300
C	−1.86408100	−0.25557300	0.79055500
C	−3.20247900	−0.29661000	1.02218000
C	−4.10401600	−0.14940400	−0.03572400
C	−3.63965100	0.04345300	−1.34966400
C	−2.31413900	0.08615300	−1.60638800
Cl	−0.78160600	−0.43551400	2.10471700
H	−3.56751200	−0.44148100	2.02563700
H	−1.95012100	0.23395000	−2.61024400
C	1.31568100	0.61826600	−0.36243000
C	2.49438400	1.25194100	−0.01429600
C	3.66900500	0.53795000	0.14809600
C	3.64591300	−0.82955700	−0.03549000
C	2.48221000	−1.49706300	−0.37630500

C	1.32637900	−0.76153000	−0.54729500
H	2.48582000	−2.56716200	−0.50123400
H	−4.35088300	0.15740100	−2.15165700
H	4.57936200	1.04530300	0.41801900
Cl	2.50903800	2.95885000	0.20679000
S	−0.19745900	1.44496700	−0.61977700
S	−0.18653600	−1.48062500	−1.03915700
Cl	5.10059200	−1.73125700	0.17515300
Cl	−5.78554800	−0.20247900	0.27338900

IM8

C	1.51516500	0.49831100	0.78928100
C	2.50180000	−0.14865600	1.52336400
C	3.68674100	−0.55096400	0.94260500
C	3.87938900	−0.32293500	−0.40775900
C	2.91935400	0.31395700	−1.16806400
C	1.74989100	0.73791900	−0.56156800
H	4.44455800	−1.05065500	1.52279200
C	−1.28154900	−1.14022500	0.70341500
C	−2.32707200	−1.74156500	−0.01481300
C	−3.33969500	−0.97228700	−0.53614900
C	−3.32569300	0.40314200	−0.39255600
C	−2.26764200	1.01072800	0.26627000
C	−1.22168200	0.27312700	0.80691100
H	−2.33650500	−2.81326800	−0.12130400
H	3.08000700	0.49584500	−2.21733100
H	−4.11606400	1.00606200	−0.80709800
Cl	−2.29828100	2.72641100	0.36834500
S	0.09103600	1.09584100	1.64320100
S	−0.17888800	−2.17138500	1.50068600
Cl	−4.64037500	−1.71741700	−1.38211700
H	2.31598900	−0.35141300	2.56609000
Cl	0.60255500	1.57886000	−1.52776400
Cl	5.34101800	−0.83733400	−1.16146100

IM9

C	1.36963100	0.57201000	−0.05575100
C	2.61998500	1.05414600	0.23524600
C	3.76367500	0.27168200	0.10681100
C	3.63211100	−1.05331500	−0.35675600
C	2.43567400	−1.59725800	−0.65988400
C	1.18588200	−0.83441300	−0.46899700
Cl	2.80867000	2.68075400	0.77372200

H	4.73076200	0.67664600	0.34756900
H	0.56249600	−0.89171100	−1.36396300
C	−1.35307000	−0.85413300	0.38469300
C	−2.51921700	−1.60557500	0.40522200
C	−3.72287800	−1.01512100	0.08671800
C	−3.78301100	0.31222400	−0.28925800
C	−2.61637600	1.04716400	−0.32754400
C	−1.37960500	0.49609800	0.01723700
H	−2.47706500	−2.65107000	0.66341600
H	2.35986900	−2.60666900	−1.02903800
H	−4.72021400	0.77241000	−0.55189700
Cl	−2.72991900	2.70534100	−0.78035000
S	−0.01560900	1.59837500	0.10404100
S	0.13955200	−1.65730500	0.82641800
Cl	−5.16964900	−1.94787600	0.12954100
Cl	5.07703100	−1.97455800	−0.55937600

IM10

C	1.51516800	0.49866500	0.78904900
C	2.50174000	−0.14811400	1.52338600
C	3.68668700	−0.55065500	0.94286500
C	3.87950800	−0.32304600	−0.40758500
C	2.91953000	0.31376100	−1.16807900
C	1.75006100	0.73798200	−0.56182700
H	4.44450100	−1.05015500	1.52322800
C	−1.28151300	−1.14010900	0.70356100
C	−2.32697400	−1.74158700	−0.01458200
C	−3.33966700	−0.97249400	−0.53615800
C	−3.32576300	0.40295500	−0.39266500
C	−2.26776400	1.01069000	0.26611500
C	−1.22171500	0.27328700	0.80673400
H	−2.33648000	−2.81332000	−0.12081500
H	3.08033100	0.49531300	−2.21738700
Cl	0.60298100	1.57882200	−1.52837200
H	2.31582600	−0.35050800	2.56617100
H	−4.11622200	1.00572100	−0.80726900
Cl	−2.29885100	2.72638800	0.36838300
S	0.09101000	1.09629400	1.64284400
S	−0.17895800	−2.17114400	1.50112000
Cl	−4.64023600	−1.71786200	−1.38200500
Cl	5.34101400	−0.83778600	−1.16107900

IM11

C	1.10889500	0.10433300	−0.93378400
C	1.72075600	−1.06493100	−0.47566600
C	3.05948500	−1.07747600	−0.12863100
C	3.79028700	0.08952800	−0.22994900
C	3.21465700	1.26378800	−0.68160000
C	1.88223300	1.25701900	−1.03429300
Cl	0.84313200	−2.53487100	−0.33434400
H	3.51954600	−1.98599700	0.22105200
H	1.41914600	2.15869400	−1.40075300
C	−1.45135400	0.86221500	0.02717500
C	−2.88081400	0.78412000	−0.38283700
C	−3.64613400	−0.24988400	−0.02637700
C	−3.14702400	−1.30857400	0.82970500
C	−1.93421700	−1.13268200	1.33825100
C	−1.07419000	−0.01597100	1.20479700
Cl	−1.00686900	2.55752700	0.29499900
H	−3.24608900	1.55960800	−1.03475400
H	−3.76121800	−2.17268900	1.02881100
H	3.80369500	2.16227900	−0.75968300
S	−0.56887100	0.13730000	−1.44557200
S	0.20140300	0.23552600	2.17471300
Cl	5.45544900	0.07227900	0.20664800
Cl	−5.27471600	−0.37470100	−0.56504900

IM12

C	−1.09739600	−0.11265000	−0.95042500
C	−1.75980500	1.04550100	−0.52083400
C	−3.10105700	1.01164200	−0.18111400
C	−3.79661100	−0.18328000	−0.24957200
C	−3.18718400	−1.36277200	−0.66160300
C	−1.87146500	−1.24062300	−0.98844000
Cl	−0.92679700	2.54454900	−0.41816200
H	−3.59694700	1.91035400	0.14310200
C	1.42619300	−0.85659700	0.05599200
C	2.85107900	−0.87357800	−0.36381700
C	3.64844000	0.15168000	−0.06791400
C	3.18776200	1.26602100	0.72090300
C	1.97089400	1.24230600	1.28449300
C	1.09408800	0.09984400	1.17264500
Cl	0.88674700	−2.50996100	0.37854200
H	3.17922400	−1.70192900	−0.96893300
H	1.63910400	2.04444500	1.92327100
H	3.85234000	2.10173600	0.86963200

H	−3.73120400	−2.29136600	−0.71348500
S	0.58040200	−0.14811200	−1.45197300
S	−0.19084800	−0.07433000	2.14821000
Cl	−5.46268000	−0.20178200	0.18445000
Cl	5.28125900	0.18901100	−0.60925600

IM13

C	1.46204400	−0.87420700	0.45123400
C	2.12907500	0.29949500	0.82418200
C	3.46260000	0.49210000	0.50178800
C	4.15154700	−0.48175300	−0.20119100
C	3.53844200	−1.66489800	−0.59695400
C	2.22913800	−1.77364200	−0.23669900
Cl	1.31718500	1.53889400	1.69216300
H	3.96053600	1.39936300	0.79854000
C	−1.06422200	−0.78330300	−0.71906200
C	−0.71391400	0.61443200	−1.04085400
C	−1.56550100	1.59792300	−0.75460200
C	−2.85560200	1.33066100	−0.18323800
C	−3.29831600	0.06978700	−0.01223700
C	−2.51042100	−1.07275900	−0.42362900
H	−3.48312600	2.16303000	0.09079900
H	−0.71073900	−1.47984500	−1.47185800
Cl	−4.87106000	−0.18473100	0.61702900
H	4.07468100	−2.42578000	−1.13981800
H	0.25968100	0.81939500	−1.45617000
S	−0.20791700	−1.25642200	0.86779500
S	−3.07327200	−2.59167700	−0.51215100
Cl	−1.15908600	3.24902100	−1.02439700
Cl	5.80759500	−0.21668900	−0.58857400

IM14

C	1.58021000	−0.99349300	−0.45098100
C	2.09881800	−0.57092400	0.77952100
C	3.38959100	−0.08116800	0.87546300
C	4.19205200	−0.02357400	−0.25254600
C	3.73013800	−0.43890700	−1.49470500
C	2.44530600	−0.89523000	−1.50424500
Cl	1.13773600	−0.63862700	2.19929200
H	3.77011700	0.24511100	1.82833400
C	−1.10255500	−0.38303000	−0.39595900
C	−0.68136300	0.93446500	−0.38425300
C	−1.60763500	1.94133200	−0.20429100

C	−2.94722700	1.66104000	−0.04122100
C	−3.35133300	0.33966500	−0.04368100
C	−2.45295900	−0.70814800	−0.21898300
H	−3.66735600	2.44940000	0.09505800
Cl	−5.03321200	0.02918500	0.18236700
H	4.35278000	−0.39409200	−2.37301100
H	0.35702500	1.18262400	−0.52795700
S	0.00130200	−1.73456800	−0.67796800
Cl	−1.08060900	3.58298100	−0.19602000
Cl	5.79950100	0.57445600	−0.10287600
S	−2.90261000	−2.40672200	−0.18369000
H	−4.14800200	−2.23050100	−0.62759100

IM15

C	−1.07466200	0.22108200	−1.05665400
C	−1.70309400	1.26497900	−0.36928200
C	−3.00364700	1.13083800	0.08753800
C	−3.69369600	−0.04847200	−0.13155900
C	−3.12066500	−1.11084400	−0.81982100
C	−1.84397600	−0.89356100	−1.24321700
Cl	−0.89350800	2.75316100	−0.08539800
H	−3.47308600	1.94358900	0.61487000
C	1.35787800	−0.78530800	−0.34895500
C	2.86112100	−0.63230400	−0.51143800
C	3.52850400	0.29606400	0.17670100
C	2.76724400	1.24287600	0.99605200
C	1.61668900	0.74861200	1.42457800
C	0.98430500	−0.36762100	1.05988900
Cl	0.96506200	−2.50853900	−0.65946700
H	3.34472600	−1.26872900	−1.23322100
H	3.06314500	2.28053700	1.02533200
H	−3.66134900	−2.02571800	−0.99832200
S	0.59225600	0.28625600	−1.62364900
Cl	−5.30577100	−0.19368800	0.45725000
Cl	5.21988700	0.52855200	−0.03312800
S	−0.03352100	−1.21045200	2.17996000
H	−0.02559500	−2.39186400	1.56001500

IM16

C	1.55206500	−0.98950400	−0.43018600
C	2.08646800	−0.57532200	0.79295100
C	3.37320900	−0.07468300	0.86290700
C	4.13827800	0.02488000	−0.28972400

C	3.64677400	−0.37253600	−1.52766600
C	2.37628300	−0.86431700	−1.50773100
Cl	1.15926900	−0.68649600	2.23485800
H	3.77735400	0.23955600	1.81022700
C	−1.11864600	−0.30830900	−0.36140500
C	−0.68891400	1.00361800	−0.33360600
C	−1.66220400	1.96375200	−0.16563900
C	−2.99905500	1.60967600	−0.02946300
C	−3.37633600	0.28649300	−0.05869900
C	−2.43793800	−0.74888400	−0.22805300
H	−3.74599200	2.37528100	0.10115000
Cl	−5.03624700	−0.10969900	0.11488900
H	4.24139500	−0.29410700	−2.42288200
H	0.34654000	1.28129100	−0.44053200
S	−2.70981100	−2.42913700	−0.27465400
Cl	−1.21829600	3.63254000	−0.12454900
Cl	5.73252100	0.65217600	−0.17707300
S	−0.08036800	−1.71442000	−0.64727000
H	−0.14955800	−2.33916700	0.54257300

IM17

C	0.96270600	−0.99004300	0.63991600
C	1.62044500	0.16790300	1.07532900
C	2.92947900	0.33339900	0.71187900
C	3.51108800	−0.64873700	−0.09604200
C	2.82293900	−1.76080700	−0.52815800
C	1.48789500	−1.99367700	−0.15535100
Cl	0.86364000	1.37596400	2.07342300
H	3.49422600	1.19014900	1.03696100
C	−1.71507800	−0.55040200	−0.49664300
C	−0.89609900	0.53037100	−1.08161000
C	−1.14259200	1.81336000	−0.80593500
C	−2.25120700	2.20979700	0.04504400
C	−3.06211900	1.24703000	0.46949600
C	−3.00560100	−0.13890500	0.20022100
H	−2.38350200	3.25085400	0.29632700
H	3.34608900	−2.46614500	−1.15887100
H	−0.05866800	0.22811100	−1.68905000
S	−4.13691800	−1.19991600	0.67810000
Cl	−0.15464100	3.06239600	−1.43495700
Cl	5.16845700	−0.41864400	−0.54638800
Cl	−1.94702800	−1.84136100	−1.65371000
S	−0.72136900	−1.31523200	0.97110400

H	−1.05395500	−0.30836900	1.80624900

IM18

C	−1.13375300	−0.01718600	−0.82172500
C	−1.89551300	1.08507900	−0.42756200
C	−3.24395700	0.90857700	−0.17252700
C	−3.81346500	−0.34972500	−0.30824500
C	−3.07751800	−1.47714300	−0.68383900
C	−1.76206600	−1.21098600	−0.88856300
Cl	−1.20496100	2.65342900	−0.35843200
H	−3.85236300	1.74526300	0.12508600
C	1.38214700	−0.70350100	0.07411100
C	2.81288000	−0.94214400	−0.33800500
C	3.73437200	0.01285900	−0.20919000
C	3.32595200	1.35414300	0.23863200
C	2.18819600	1.34407900	0.88418000
C	1.28375900	0.36019800	1.19825700
Cl	0.67850100	−2.22166100	0.59700700
H	3.04784700	−1.90328500	−0.76255000
H	3.83359700	2.22548800	−0.14708700
H	−3.53198300	−2.45389000	−0.72453500
S	0.51813200	−0.02239500	−1.40307100
S	0.28403400	0.25660400	2.48842000
Cl	−5.48609400	−0.53809800	0.06443600
Cl	5.35737900	−0.21992900	−0.72240200

IM19

C	−1.73830000	−1.32524300	−0.88595800
C	−1.88203500	−2.00284600	0.37878400
C	−0.79901200	−2.27542500	1.12142900
C	0.53301400	−1.93104300	0.68543800
C	0.62459800	−0.86360500	−0.39804100
C	−0.57369600	−0.82329500	−1.28651600
H	−2.86431200	−2.32477000	0.68559700
H	−0.31323800	0.41886300	2.21168600
C	−0.37260200	0.86731600	1.23366700
C	−1.41011900	1.62669300	0.88173300
C	0.78243900	0.57818800	0.28306400
C	−1.51893900	2.02722800	−0.52508200
C	0.85799000	1.72623800	−0.72851000
Cl	2.24499000	0.59089400	1.29937500
C	−0.34432000	2.13221300	−1.11464600
H	−0.46764300	−0.32351900	−2.23421900

H	−2.47492500	1.95567300	−1.02375600
S	1.82148500	−2.65390700	1.34920100
Cl	−2.71528600	1.92410600	1.96278900
Cl	−3.14389500	−1.18900200	−1.87129600
H	−0.87873800	−2.85417100	2.02731800
Cl	2.05028200	−1.10431200	−1.43459600
S	2.27283400	2.58244600	−1.25193600
H	3.12208700	2.08413500	−0.35167200

IM20

C	−1.85447500	1.59142100	1.02738400
C	−3.05516700	0.97569900	0.74322600
C	−3.05335400	−0.18196100	−0.00872300
C	−1.87280700	−0.74119400	−0.49747600
C	−0.67381900	−0.08130400	−0.21350100
C	−0.66963700	1.06941500	0.55470900
H	−3.98458700	1.38497300	1.10126500
Cl	−4.58715000	−0.91017100	−0.32639400
H	4.14488800	−0.36816700	1.07505600
C	3.19594900	−0.43639500	0.56794900
C	2.29614000	−1.49666000	0.93642800
C	2.88435700	0.45630100	−0.39147300
C	1.10735300	−1.63044000	0.35688700
C	1.63003300	0.40868700	−1.10915300
Cl	4.01855600	1.68394600	−0.77444700
C	0.64774400	−0.66453000	−0.68225200
H	0.44421600	−1.23023000	−1.59517900
H	0.25982500	1.56993100	0.77432500
H	0.43313700	−2.42560200	0.63501000
S	1.24061800	1.42645300	−2.31256200
Cl	2.83949000	−2.58234500	2.16078600
Cl	−1.84153700	3.02741200	1.97857600
S	−1.80150700	−2.23206000	−1.42295400
H	−3.11625800	−2.37572200	−1.58111100

IM21

C	−2.76301300	−0.49387200	−1.12553700
C	−3.41429500	0.75322900	−0.82575800
C	−2.94053900	1.56605100	0.13484100
C	−1.76898000	1.25545800	0.89873900
C	−1.05989600	−0.07115400	0.60804300
C	−1.68467900	−0.90292600	−0.46305200
H	−4.29911100	1.01800300	−1.38277600

H	4.02955600	0.73516000	−0.98602600
C	3.01673700	0.56626500	−0.66219500
C	2.07768000	1.56811300	−0.70752900
C	2.63663600	−0.67492200	−0.19098600
C	0.79249000	1.34274400	−0.26255400
C	1.33496300	−0.95816900	0.23150900
Cl	3.87328700	−1.87988000	−0.16455800
C	0.40937500	0.10241100	0.22702600
H	−1.20667700	−1.84400600	−0.68409500
H	0.08760100	2.15206100	−0.31426800
S	−1.23374400	2.25788200	2.06227200
Cl	2.51088800	3.12030300	−1.31333800
Cl	−3.44752500	−1.45054000	−2.38535700
Cl	−1.25082000	−0.98823100	2.16148900
H	−3.43934400	2.49171400	0.37176500
S	0.89203300	−2.58998300	0.73577800
H	1.81739200	−3.18678200	−0.01869600

IM22

C	−1.26928000	1.21102400	−1.36044600
C	−2.23207000	0.76301700	−0.38670800
C	−2.21367800	−0.50635200	0.05702400
C	−1.24051800	−1.45222800	−0.45772000
C	0.01092700	−0.83255400	−1.02208500
C	−0.22016000	0.46736700	−1.70605100
H	−2.99725200	1.44668100	−0.05670600
H	0.46188000	−1.55025400	−1.70380600
Cl	−3.42330400	−1.04480400	1.14412500
H	−0.00215700	−0.14290400	1.97820900
C	0.63188600	0.29630700	1.22494000
C	0.95661100	1.58955800	1.26216600
C	1.10863200	−0.62490400	0.11179700
C	1.64011200	2.15032400	0.09283100
C	2.41676800	−0.05886900	−0.43945500
Cl	1.43362100	−2.19866100	0.89032100
C	2.39447200	1.26242100	−0.52765600
H	0.50242400	0.81344500	−2.42575300
H	1.31399300	3.09811400	−0.31107700
S	−1.45727100	−3.05581200	−0.44643900
Cl	0.39032000	2.63066200	2.50918200
Cl	−1.53267400	2.76833400	−2.05006500
S	3.85906100	−0.93673800	−0.85693700
H	3.63489400	−2.01898800	−0.10926100

IM23

C	−2.76097300	1.27824700	−0.88336600
C	−3.53127800	0.39284300	−0.15235600
C	−2.90291900	−0.54395600	0.64024000
C	−1.49244100	−0.62608000	0.72868800
C	−0.74341600	0.30053600	−0.04747400
C	−1.37420500	1.23750100	−0.83681500
H	−4.60630800	0.43425700	−0.19830700
H	−0.42338100	−1.56577000	−1.51800700
Cl	−3.89802400	−1.61374600	1.53135300
H	4.58360700	0.48938300	0.23740100
C	3.50936000	0.44877000	0.17405200
C	2.72384300	1.38952500	0.80440200
C	2.89525000	−0.55269700	−0.55150700
C	1.34762500	1.32517200	0.71969300
C	1.51102900	−0.64887300	−0.66094800
Cl	3.89212300	−1.71295200	−1.34339200
C	0.73807300	0.30658200	0.00585600
H	−0.79428400	1.93401300	−1.42016300
H	0.74186800	2.05882000	1.22646100
S	0.84804700	−1.95504100	−1.62468800
S	−0.70781800	−1.75920300	1.72230200
Cl	3.46803700	2.65578800	1.70650300
Cl	−3.53654000	2.44925100	−1.86693300

IM24

C	3.13626600	1.33438100	0.03305700
C	3.58226700	0.04230300	−0.20084100
C	2.65952900	−0.98170000	−0.30910500
C	1.30813200	−0.72094800	−0.18048400
C	0.87532900	0.59159000	0.04517600
C	1.79399700	1.62697200	0.15398800
H	4.63488200	−0.16511400	−0.29473000
H	−0.06138300	−0.82241100	2.43123000
Cl	3.19426000	−2.58647000	−0.61660000
H	−4.31597600	0.22682900	−0.74613300
C	−3.28532000	0.32292300	−0.44710700
C	−2.71074200	1.60602400	−0.32156600
C	−2.53193700	−0.77851400	−0.23192000
C	−1.34489700	1.77235700	−0.12449900
C	−1.16377500	−0.66893200	0.31987200
Cl	−3.18167200	−2.34907900	−0.42312200

C	−0.55973600	0.67061100	0.07596800
H	1.47276300	2.63893600	0.33969400
H	−0.90991900	2.75555100	−0.20880600
S	0.02588100	−1.89332800	−0.32058400
S	−1.37289000	−0.88557400	2.17878600
Cl	4.29699400	2.60166400	0.17861600
Cl	−3.71093000	2.97660500	−0.55817100

IM25

C	−3.34345700	0.50818900	−0.53171400
C	−3.88965300	−0.22442600	0.51130100
C	−3.04278700	−0.82002700	1.41640900
C	−1.64574000	−0.70495300	1.30933200
C	−1.11350700	0.04017300	0.22737200
C	−1.96914900	0.64059400	−0.67567900
H	−4.95925100	−0.31692200	0.60503500
H	−0.69064300	−2.11300100	−0.72667600
H	0.22897600	2.24413600	0.75141900
C	0.87641800	1.46690000	0.37885900
C	2.22937800	1.70252400	0.24134900
C	0.34667000	0.22700500	0.06040400
C	3.07035900	0.71205400	−0.21881400
C	1.17406200	−0.78902200	−0.42848600
C	2.53341000	−0.51576300	−0.55028700
H	−1.56988500	1.20953000	−1.50016500
H	4.12764300	0.88646400	−0.32580900
S	0.60463000	−2.37829500	−0.90352400
S	−0.64216100	−1.44162200	2.47616600
Cl	2.87636700	3.24702400	0.65117900
Cl	−4.38190200	1.26429000	−1.67104400
H	−3.44264900	−1.38860300	2.24079900
Cl	3.59672800	−1.73851600	−1.13584800

IM26

C	3.60307100	0.33340100	−0.09431700
C	3.75881500	−1.00504200	−0.43033300
C	2.64698700	−1.81381100	−0.57874500
C	1.38852000	−1.27207500	−0.39219700
C	1.23459200	0.07762100	−0.05485800
C	2.35494400	0.88662300	0.09489100
H	4.74891300	−1.40813300	−0.56726000
H	0.10438300	−1.28849700	2.25155000
H	−0.07045200	2.58022000	−0.05247700

C	−0.69694600	1.70278600	−0.02684200
C	−2.07372300	1.83677500	−0.16623100
C	−0.15026500	0.45090100	0.04542500
C	−2.90674800	0.71550200	−0.36571400
C	−1.01438000	−0.75233800	0.21205200
C	−2.39177500	−0.53134100	−0.27820700
H	2.25716400	1.92582000	0.36562700
H	−3.94399000	0.85997200	−0.61904200
S	−1.20235800	−1.08045100	2.05873500
S	−0.12424000	−2.12716400	−0.58309200
Cl	−2.77389200	3.39974500	−0.23796500
Cl	5.00562900	1.32124000	0.10029600
H	2.76862900	−2.85374700	−0.83718000
Cl	−3.35865700	−1.91320900	−0.56474600

IM27

C	−0.90555000	1.27624400	−1.52945500
C	−2.01642700	1.10113800	−0.62656400
C	−2.25179900	−0.09076000	−0.05159200
C	−1.40973800	−1.24059000	−0.34013800
C	−0.04465300	−0.91574900	−0.88482700
C	0.01828100	0.33312500	−1.69487900
H	−2.68211000	1.93109900	−0.45423200
H	0.31559900	−1.76698300	−1.46201900
Cl	−3.61995700	−0.28388500	0.96138500
H	−0.26861900	0.01408700	1.86316300
C	0.60108700	0.24407800	1.26906000
C	1.24511600	1.40065400	1.41891100
C	1.02602000	−0.78509900	0.27735400
C	2.44756100	1.70499900	0.66635600
C	2.37254900	−0.51381700	−0.41499700
Cl	1.16666500	−2.32505300	1.16268400
C	2.90882600	0.76056800	−0.14796000
H	0.84356100	0.46910800	−2.37493600
H	2.93275300	2.65980100	0.79985800
S	3.06656500	−1.57549600	−1.43086400
S	−1.87450100	−2.77116000	−0.10338500
Cl	0.70293800	2.58719400	2.54073700
Cl	−0.84740100	2.76237100	−2.39842000

IM28

C	−2.76392100	−0.46468500	−1.12889400
C	−3.41852500	0.77070100	−0.78738500

C	−2.94079500	1.56061100	0.18997700
C	−1.76158400	1.23475300	0.93610600
C	−1.04907200	−0.07606300	0.59707800
C	−1.67748200	−0.88573200	−0.48776800
H	−4.30914200	1.04630400	−1.32974500
H	4.04260400	0.66992100	−1.00714600
C	3.02986200	0.52141600	−0.67255400
C	2.10896000	1.54355100	−0.70985700
C	2.62758600	−0.70912800	−0.19533600
C	0.81423400	1.34752300	−0.26821100
C	1.32128900	−0.95982700	0.23449800
Cl	3.78758000	−1.98116100	−0.18491100
C	0.41000600	0.11278000	0.20832800
H	−1.19184900	−1.81633400	−0.73652200
H	0.12554800	2.17278400	−0.30652600
S	−1.21117400	2.20589300	2.11789700
S	0.91268600	−2.57043300	0.73454900
Cl	2.57574400	3.08950100	−1.30426200
Cl	−3.45431000	−1.38657700	−2.40987700
Cl	−1.18272300	−1.05665100	2.12201400
H	−3.44165000	2.47765200	0.45453000

IM29

C	−1.88668000	1.48591300	1.09524000
C	−3.09513600	0.89600800	0.76386000
C	−3.09040600	−0.22006600	−0.03892500
C	−1.88691200	−0.78459900	−0.53907800
C	−0.67721900	−0.12199100	−0.19589700
C	−0.68542700	0.98540500	0.62162100
H	−4.02269700	1.30226200	1.12954300
Cl	−4.60516100	−0.91669900	−0.42585500
H	4.18679100	−0.32472000	0.99980800
C	3.22395200	−0.40372000	0.52120400
C	2.34663300	−1.47278600	0.91722400
C	2.87611100	0.48177400	−0.43282200
C	1.14517300	−1.62148800	0.36714100
C	1.59688700	0.42722000	−1.10519800
Cl	3.98822500	1.71717900	−0.85316400
C	0.65329500	−0.67038800	−0.66765700
H	0.43665700	−1.26307600	−1.56026300
H	0.23679600	1.47793800	0.88598000
H	0.48955900	−2.43148900	0.64640500

S	−1.89221100	−2.18847500	−1.48782700
S	1.14839500	1.46807000	−2.26981600
Cl	2.93842600	−2.55650600	2.12034400
Cl	−1.88311800	2.86648900	2.11047100

IM30

C	−1.52563000	1.32146300	1.09148600
C	−1.70827300	2.12359100	−0.09365100
C	−0.68035400	2.34381300	−0.92564100
C	0.64026800	1.81345700	−0.68405300
C	0.75229800	0.71942000	0.37492700
C	−0.38020000	0.69742300	1.34762400
H	−2.67306700	2.57094000	−0.27141300
H	−0.28907900	−0.16153600	−2.22041600
C	−0.39975400	−0.73369800	−1.31410600
C	−1.53157000	−1.38395000	−1.04745300
C	0.75855200	−0.71611600	−0.36550100
C	−1.69695000	−2.17791300	0.15860800
C	0.68847500	−1.82135000	0.70028000
Cl	2.23455500	−0.91711900	−1.32230500
C	−0.61698100	−2.31674100	0.91614900
H	−0.24066300	0.12357900	2.24907400
H	−2.65262500	−2.62498900	0.38313400
S	1.96093800	−2.38994500	1.52618600
S	1.90940800	2.35826600	−1.52964500
Cl	−2.86550300	−1.32319000	−2.13304100
Cl	−2.85169500	1.22236900	2.18434700
H	−0.78760000	2.99475400	−1.77826900
Cl	2.24253800	0.92310000	1.31460000

IM31

C	−2.39184500	−0.98182200	−1.04448400
C	−2.04766700	0.10847800	−1.97952400
C	−1.86710600	1.24634400	−1.34081400
C	−1.77860100	1.46834400	−0.03416100
C	−1.06425100	0.29398800	0.62920100
C	−1.90831400	−0.92252700	0.19445900
H	−1.80102500	−0.10841900	−3.00750600
H	4.16417400	0.25507500	−0.75202700
C	3.12569900	0.24378700	−0.46829900
C	2.35191600	1.38254700	−0.56185300
C	2.54179100	−0.90948100	0.00604300

C	1.00906800	1.37368700	−0.21786800
C	1.18093800	−0.96702400	0.40356700
Cl	3.54444700	−2.29371100	0.11539700
C	0.41154700	0.21966500	0.23936700
H	−2.08523800	−1.69138700	0.92205800
H	0.44250800	2.28235500	−0.32255700
S	0.55682900	−2.38450000	1.09610900
Cl	3.06251900	2.83731400	−1.12620600
Cl	−3.28174000	−2.34036100	−1.61269000
Cl	−1.10842400	0.37887000	2.42648800
S	−2.64592500	2.73863700	0.75107800
H	−2.35954600	2.36436100	2.00041800

IM32

C	−1.72879100	−1.37469400	−0.84183100
C	−1.86484300	−2.02302800	0.45124000
C	−0.73347100	−2.22615000	1.11512800
C	0.58445300	−1.92291400	0.71224600
C	0.64236500	−0.84785100	−0.37745700
C	−0.57187200	−0.85506300	−1.25206400
H	−2.83869000	−2.31940300	0.80808400
H	−0.32959100	0.42369300	2.22162100
C	−0.39204700	0.86330900	1.23986500
C	−1.45094800	1.58320800	0.86739800
C	0.77093400	0.59988800	0.29886300
C	−1.55510200	2.00196400	−0.52913100
C	0.82993900	1.73292900	−0.72492400
Cl	2.23271900	0.62911500	1.31755500
C	−0.37922000	2.12901500	−1.12947300
H	−0.48689500	−0.36732400	−2.20810600
H	−2.51403700	1.96567800	−1.02517900
S	1.86695900	−2.67053800	1.36555500
Cl	−2.78263900	1.83162600	1.92797100
Cl	−3.13838600	−1.27916800	−1.82464000
Cl	2.06682400	−1.07641800	−1.41265300
S	2.24086500	2.57553500	−1.27345300
H	3.10083500	2.06608000	−0.38917400

IM33

C	−2.78135900	−0.45400900	−1.13322500
C	−3.39100900	0.83950800	−0.90449600
C	−2.85665700	1.59561500	0.05100200
C	−1.76383600	1.31782200	0.88883300

C	−1.07674000	−0.03840400	0.62646700
C	−1.72960900	−0.87434000	−0.43028500
H	−4.24131400	1.14317600	−1.49554700
H	4.01399200	0.67148700	−1.00625700
C	3.00093700	0.52328800	−0.67310400
C	2.08178300	1.54337000	−0.71287800
C	2.60176200	−0.70880000	−0.19323700
C	0.79534700	1.34420400	−0.25769500
C	1.29890800	−0.96536300	0.24151900
Cl	3.81543500	−1.93637700	−0.16936000
C	0.39177300	0.11147600	0.23455100
H	−1.28566800	−1.83977300	−0.61463300
H	0.10673500	2.16805300	−0.30498700
S	−1.28195700	2.33707300	2.06096400
Cl	2.54059500	3.08538900	−1.32478500
Cl	−3.47559200	−1.43510300	−2.36726200
Cl	−1.26603600	−0.91424400	2.19812600
S	0.83148400	−2.58316200	0.76762000
H	1.74119700	−3.20602000	0.01515400

IM34

C	2.73258600	1.33102200	0.88587000
C	3.51607800	0.43950700	0.18283600
C	2.90120900	−0.51694400	−0.59938900
C	1.51424400	−0.61521400	−0.70002400
C	0.74090700	0.29622800	0.03108400
C	1.35590500	1.26335300	0.81243200
H	4.59084400	0.48454400	0.23575000
H	0.43060600	−1.53659400	1.51553300
Cl	3.89588500	−1.61360800	−1.47843600
H	−4.59083400	0.48457300	−0.23577000
C	−3.51606900	0.43952900	−0.18284600
C	−2.73256400	1.33104800	−0.88585900
C	−2.90121400	−0.51693800	0.59937000
C	−1.35588500	1.26337100	−0.81240600
C	−1.51425100	−0.61521800	0.70002200
Cl	−3.89590600	−1.61360300	1.47839900
C	−0.74090000	0.29623400	−0.03106000
H	−0.43063500	−1.53662700	−1.51540500
H	0.74976400	1.95995400	1.36869200
H	−0.74973400	1.95997400	−1.36865200
S	0.84583600	−1.86022100	−1.73575600
S	−0.84587200	−1.86023900	1.73575800

Cl	3.48160800	2.53522200	1.86377400
Cl	−3.48156800	2.53526400	−1.86375700

IM35

C	−1.61555800	1.56516700	0.69565700
C	−1.66463500	1.83720500	−0.74885900
C	−0.46827000	2.04028900	−1.26390300
C	0.72999900	1.83629800	−0.73234900
C	0.69856600	0.72463300	0.31137100
C	−0.52799800	0.96983100	1.18474400
H	−2.56947700	1.61998700	−1.29780600
H	−0.48295900	−0.61197100	−2.19939000
C	−0.52807900	−0.96990300	−1.18488800
C	−1.61562900	−1.56512800	−0.69565500
C	0.69856700	−0.72471600	−0.31163000
C	−1.66465200	−1.83733800	0.74882600
C	0.72992700	−1.83631700	0.73212500
Cl	2.11498300	−0.80801200	−1.39960400
C	−0.46824700	−2.04024900	1.26384600
H	−0.48271500	0.61212900	2.19931500
H	−2.56960600	−1.62052500	1.29773400
Cl	−3.02939400	−1.77715400	−1.65295400
Cl	−3.02913500	1.77740300	1.65312200
Cl	2.11501400	0.80781100	1.39936200
S	2.05250900	−2.89273900	1.10575200
H	2.84160000	−2.54062000	0.08862400
S	2.05262800	2.89287600	−1.10537500
H	2.84205600	2.53937300	−0.08897200

IM36

C	−2.36159100	−0.88119400	−1.23253900
C	−2.14384400	0.39031700	−1.94642400
C	−2.02045000	1.39562900	−1.10567500
C	−1.87150500	1.38380800	0.21275200
C	−1.07686700	0.15124800	0.64152500
C	−1.83220600	−1.02210100	−0.01943600
H	−1.91350900	0.39192500	−3.00076500
H	4.09977900	0.52549000	−0.83580500
C	3.07141800	0.44889500	−0.52614500
C	2.22605200	1.53147700	−0.58651900
C	2.58174400	−0.74289200	−0.02848200
C	0.90813600	1.41927200	−0.19530800
C	1.25573700	−0.89816100	0.37812200

Cl	3.70444200	−2.04897400	0.07537000
C	0.40105500	0.21066000	0.25480400
H	−1.90015100	−1.94482500	0.52482200
H	0.27101900	2.28293100	−0.26269200
Cl	2.81776900	3.04349800	−1.16132000
Cl	−3.15067800	−2.18354700	−2.03626700
Cl	−1.15223900	−0.09946000	2.42534400
S	0.71035900	−2.39865100	1.12222500
H	1.46117500	−3.20372800	0.36886600
S	−2.71668800	2.50163700	1.22706500
H	−2.47111200	1.88289200	2.38370700

2,4-DCTP

C	0.53672800	0.61797800	0.00054600
C	−0.82054200	0.89072700	0.00286600
C	−1.72164300	−0.15212700	0.00015200
C	−1.28005800	−1.46275300	0.00073200
C	0.07421900	−1.71592900	0.00665400
C	1.01448500	−0.68856900	0.00623900
H	−1.16237500	1.91207000	0.00289200
H	−1.98855900	−2.27444300	0.00076000
H	0.41949600	−2.73843100	0.01511400
S	2.70364800	−1.15652000	−0.01202100
H	3.21265100	0.06401300	0.15216900
Cl	1.61510200	1.96716600	−0.00316700
Cl	−3.41267400	0.18607700	−0.00164100

R1

C	0.76623500	0.89756300	0.00000400
C	−0.58303700	0.61291700	0.00000800
C	−1.05442000	−0.72079400	0.00000100
C	−0.07879500	−1.73869600	0.00001000
C	1.26657500	−1.46886400	0.00001600
C	1.67957100	−0.14326000	0.00000400
H	1.10615600	1.91942800	0.00001300
H	−0.42786700	−2.75888200	−0.00000300
H	1.99422900	−2.26329400	0.00003600
S	−2.70302700	−1.13092600	−0.00001100
Cl	−1.67530000	1.93114700	0.00000300
Cl	3.35760300	0.21969800	−0.00001100

R2

C	1.29329800	−1.50694100	0.00048000

C	−0.05495700	−1.67603200	0.00204000
C	−1.03808000	−0.71990400	0.00277500
C	−0.54611100	0.58792400	−0.00110100
C	0.81461400	0.85389700	0.00119000
C	1.72441900	−0.18479700	0.00057000
H	1.99067100	−2.32874100	0.00062700
H	1.15618300	1.87495800	0.00120100
Cl	−1.62344900	1.93789200	−0.00245800
Cl	3.41240300	0.16424200	−0.00008800
S	−2.71815300	−1.21382600	−0.00588500
H	−3.22770000	0.01383300	0.09988700

DR

C	0.75765500	0.86421600	0.00000100
C	−0.59325500	0.59336800	0.00000600
C	−1.07200900	−0.74511300	−0.00000100
C	−0.06747200	−1.69858500	0.00000700
C	1.27864700	−1.51231500	0.00000900
C	1.67950400	−0.17860500	−0.00000200
H	1.10342800	1.88391700	0.00000100
H	1.99556800	−2.31678800	0.00001200
S	−2.70390800	−1.20407300	−0.00000500
Cl	−1.69173300	1.90472500	0.00000200
Cl	3.35438600	0.19881800	−0.00000400

1,3,7-TCTA

C	0.24871700	−1.79721800	0.00000000
C	0.28873500	−3.18712300	0.00000000
C	1.48085000	−3.87943800	0.00000000
C	2.66272100	−3.16368500	0.00000000
C	2.64901000	−1.78541900	0.00000000
C	1.44292600	−1.09501200	0.00000000
C	−1.19542700	0.60980000	0.00000000
C	−2.37993300	1.34632200	0.00000000
C	−2.40259500	2.72633300	0.00000000
C	−1.19984900	3.39978700	0.00000000
C	−0.00750400	2.71010200	0.00000000
C	0.00000000	1.32148800	0.00000000
H	0.92495400	3.25224000	0.00000000
H	3.58104600	−1.24204000	0.00000000
S	1.62018700	0.64900900	0.00000000
S	−1.37798400	−1.13488600	0.00000000

H	−0.64043600	−3.73782100	0.00000000
H	−3.34012300	3.25481500	0.00000000
Cl	−3.89153000	0.51964700	0.00000000
Cl	−1.18781200	5.12153700	0.00000000
H	1.49242500	−4.95648600	0.00000000
Cl	4.17234100	−3.99602500	0.00000000

1,3,8-TCTA

C	−0.91906700	−1.45097900	0.00000000
C	−1.96723900	−2.36438700	0.00000000
C	−1.71417500	−3.71929000	0.00000000
C	−0.41625600	−4.19385400	0.00000000
C	0.61917400	−3.28435900	0.00000000
C	0.38669800	−1.91391700	0.00000000
C	0.00000000	1.20088800	0.00000000
C	−0.19616200	2.58201300	0.00000000
C	0.84645900	3.48579500	0.00000000
C	2.13509800	2.99708800	0.00000000
C	2.37244500	1.64001500	0.00000000
C	1.31368400	0.74215100	0.00000000
H	3.38703000	1.27381300	0.00000000
H	1.63731900	−3.64446900	0.00000000
S	1.83779600	−0.93144400	0.00000000
S	−1.45574200	0.22208500	0.00000000
H	−2.98711200	−2.01181600	0.00000000
H	0.64833200	4.54376000	0.00000000
H	−0.22039800	−5.25304800	0.00000000
Cl	−1.80078900	3.21088500	0.00000000
Cl	3.46237400	4.09408500	0.00000000
Cl	−3.03464300	−4.82764500	0.00000000

1,3,6,8-TeCTA

C	−1.24973200	−0.95113500	0.00000000
C	−2.37072300	−1.78046000	0.00000000
C	−2.27938900	−3.15695800	0.00000000
C	−1.02528700	−3.72938200	0.00000000
C	0.10662700	−2.94337900	0.00000000
C	0.00000000	−1.55957500	0.00000000
C	−0.00006000	1.55968500	0.00000000
C	−0.10665000	2.94360300	0.00000000
C	1.02528500	3.72942700	0.00000000
C	2.27942100	3.15686700	0.00000000
C	2.37071800	1.78043000	0.00000000

C	1.24954900	0.95129800	0.00000000
H	1.08046500	−3.40732000	0.00000000
S	1.56932200	−0.76993100	0.00000000
S	−1.56937700	0.77008700	0.00000000
H	−3.17075200	−3.76035100	0.00000000
H	−1.08049800	3.40751400	0.00000000
H	3.17074500	3.76031800	0.00000000
Cl	−3.94472700	−1.07921200	0.00000000
Cl	−0.87197100	−5.44387500	0.00000000
Cl	0.87239500	5.44398600	0.00000000
Cl	3.94444200	1.07879600	0.00000000

1,3,7,9-TeCTA

C	0.00000000	1.46563700	0.00000000
C	0.24953300	2.83826000	0.00000000
C	−0.75835500	3.78088100	0.00000000
C	−2.06519700	3.34412300	0.00000000
C	−2.35519400	1.99691900	0.00000000
C	−1.32990800	1.06136300	0.00000000
C	0.83307300	−1.20799600	0.00000000
C	1.81498700	−2.19980900	0.00000000
C	1.51511300	−3.54699800	0.00000000
C	0.18963800	−3.92422700	0.00000000
C	−0.81034100	−2.97652700	0.00000000
C	−0.49232900	−1.62566700	0.00000000
H	−3.38298500	1.66950300	0.00000000
S	−1.90751000	−0.59211900	0.00000000
S	1.42861300	0.44348700	0.00000000
H	−0.51942200	4.83040800	0.00000000
Cl	1.87465300	3.41100300	0.00000000
Cl	−3.34853400	4.49104000	0.00000000
H	2.30529600	−4.27789500	0.00000000
H	−1.84320000	−3.28721900	0.00000000
Cl	3.47906200	−1.75228600	0.00000000
Cl	−0.21950200	−5.59637100	0.00000000

2,4,7,9-TeCTA

C	−1.24973200	−0.95113500	0.00000000
C	−2.37072300	−1.78046000	0.00000000
C	−2.27938900	−3.15695800	0.00000000
C	−1.02528700	−3.72938200	0.00000000
C	0.10662700	−2.94337900	0.00000000
C	0.00000000	−1.55957500	0.00000000

C	−0.00006000	1.55968500	0.00000000
C	−0.10665000	2.94360300	0.00000000
C	1.02528500	3.72942700	0.00000000
C	2.27942100	3.15686700	0.00000000
C	2.37071800	1.78043000	0.00000000
C	1.24954900	0.95129800	0.00000000
H	1.08046500	−3.40732000	0.00000000
S	1.56932200	−0.76993100	0.00000000
S	−1.56937700	0.77008700	0.00000000
H	−3.17075200	−3.76035100	0.00000000
H	−1.08049800	3.40751400	0.00000000
H	3.17074500	3.76031800	0.00000000
Cl	−3.94472700	−1.07921200	0.00000000
Cl	−0.87197100	−5.44387500	0.00000000
Cl	0.87239500	5.44398600	0.00000000
Cl	3.94444200	1.07879600	0.00000000

2,6,8-TCDT

C	−2.28436800	1.62754600	0.00004500
C	−3.07369100	0.48265400	−0.00006700
C	−2.46492300	−0.75239700	−0.00013100
C	−1.08203000	−0.84537800	−0.00007000
C	−0.30221400	0.31413500	0.00004300
C	−0.91005700	1.56316700	0.00009900
H	−4.14787500	0.55854700	−0.00010000
Cl	−3.41473000	−2.18648200	−0.00023000
H	4.69010700	−1.40928000	−0.00002100
C	3.68037700	−1.03278200	−0.00001600
C	3.46050000	0.34172000	0.00008000
C	2.61111800	−1.90133000	−0.00008100
C	2.19014400	0.86888500	0.00011500
C	1.32497000	−1.38196900	−0.00004800
C	1.10605800	−0.00184500	0.00005300
H	−0.32614300	2.46897600	0.00018600
H	2.04795500	1.93779200	0.00018700
S	−0.14887300	−2.30464300	−0.00007200
Cl	−3.06413600	3.16673700	0.00011800
Cl	4.82018400	1.40743200	0.00016400
H	2.78022300	−2.96686400	−0.00012500

2,4,6,8-TeCDT

C	−2.93915200	1.38242200	0.00012200
C	−3.45994000	0.09295700	0.00006600

C	−2.59851000	−0.98130000	−0.00001400
C	−1.22757700	−0.77248500	−0.00008400
C	−0.72132100	0.52958700	−0.00001900
C	−1.58394700	1.61813800	0.00009600
H	−4.52511700	−0.06581600	0.00005900
Cl	−3.21767100	−2.58526800	−0.00017200
H	4.52512200	−0.06581000	0.00012800
C	3.45994800	0.09296400	0.00010500
C	2.93915400	1.38242100	0.00011500
C	2.59850700	−0.98130000	0.00003500
C	1.58394100	1.61814000	0.00006700
C	1.22757800	−0.77248200	−0.00004600
Cl	3.21769200	−2.58526800	−0.00004600
C	0.72131300	0.52958600	−0.00000500
H	−1.21042700	2.62901100	0.00021600
H	1.21043000	2.62901400	0.00014400
S	−0.00000700	−1.99811500	−0.00044700
Cl	−4.03436500	2.71501100	0.00021400
Cl	4.03435300	2.71502700	0.00023700
